# X-Ray Micro- and Nanodiffraction Imaging on Human Mesenchymal Stem Cells and Differentiated Cells

**DOI:** 10.1016/j.bpj.2015.12.017

**Published:** 2016-02-02

**Authors:** Marten Bernhardt, Marius Priebe, Markus Osterhoff, Carina Wollnik, Ana Diaz, Tim Salditt, Florian Rehfeldt

**Affiliations:** 1 Institut für Röntgenphysik, Georg-August-Universität Göttingen, Göttingen, Germany; 2 Drittes Physikalisches Institut - Biophysik, Georg-August-Universität Göttingen, Göttingen, Germany; 3 Paul Scherrer Institut, Villigen, Switzerland

## Abstract

Adult human mesenchymal stem cells show structural rearrangements of their cytoskeletal network during mechanically induced differentiation toward various cell types. In particular, the alignment of acto-myosin fibers is cell fate-dependent and can serve as an early morphological marker of differentiation. Quantification of such nanostructures on a mesoscopic scale requires high-resolution imaging techniques. Here, we use small- angle x-ray scattering with a spot size in the micro- and submicrometer range as a high-resolution and label-free imaging technique to reveal structural details of stem cells and differentiated cell types. We include principal component analysis into an automated empirical analysis scheme that allows the local characterization of oriented structures. Results on freeze-dried samples lead to quantitative structural information for all cell lines tested: differentiated cells reveal pronounced structural orientation and a relatively intense overall diffraction signal, whereas naive human mesenchymal stem cells lack these features. Our data support the hypothesis of stem cells establishing ordered structures along their differentiation process.

## Introduction

Human mesenchymal stem cells (hMSCs) provide a source for a broad spectrum of cell types for regeneration (1, 2). Induced by biochemical signals (3) as well as by the mechanical properties of the surrounding tissue (4, 5, 6, 7), multipotent hMSCs undergo differentiation toward terminally differentiated and thus specialized cells. Besides changes in gene regulation, hMSCs also reorganize their acto-myosin network during this process. These structural changes are cell fate-dependent, ranging from randomly oriented to parallel fiber bundles (4). An intimate understanding of the structural change as a function of cell fate is essential to improve stem cell treatment—possibly without the need of external biochemical induction—and might enable new approaches in medicine (8, 9).

Visible light microscopy with its specific labeling capability is arguably the most important tool to visualize a specific protein network, fundamentally enhanced by the recent superresolution capabilities, such as stimulated emission depletion microscopy (10, 11) or stochastic switching microscopy (12, 13, 14). However, the strength of fluorescence microscopy—specific labeling on a molecular level—is also a weakness, as unlabeled cellular constituents remain invisible. Therefore, complementary imaging methods capable to probe the native unlabeled density distribution are needed. To this end, small-angle x-ray scattering (SAXS), which is commonly applied to study homogeneous macromolecular structures in macroscopic solutions or suspensions (15, 16, 17, 18), has been recently introduced in cellular imaging, by focusing the beam down to the subcellular scales (19, 20, 21, 22). In this manner, every diffraction pattern of a given scan over the cells contains the corresponding local structural information. Depending on the range of scattering vectors, also known as momentum transfer, structures down to molecular scales become accessible (22). Recent proof-of-concept experiments and applications included scanning nanodiffraction of keratin-enriched human carcinoma cells, studied in a correlative manner by visible light fluorescence (20), and bacteria (19, 23). The latter were enhanced by coherent x-ray imaging providing an inversion of the local diffraction image and hence a superresolution image of the electron density distribution with a resolution better than the beam size. This method can even be applied to living cells with some restrictions, concerning in particular radiation damage and signal/noise (21, 22). A nano-SAXS study of *Dictyostelium discoideum* revealed pronounced anisotropic scattering on the perimeter of the cell with a predominant orientation of diffraction streaks perpendicular to the plasma membrane, attributed to actin fiber bundles oriented parallel to the membrane, also known as the actomyosin cortex (22). Similar streaklike diffraction patterns were observed in frozen-hydrated suspensions, i.e., thin vitrified films, of in vitro F-actin, cross linked with *α*-actinin, indicating highly ordered bundles of filaments.

In this work, we use micro- and nanofocus SAXS, in the following denoted as micro- and nano-SAXS, to study naive hMSCs, biochemically induced hMSCs driven toward the myogenic lineage, in the following denoted as muscle-induced hMSCs (mi-hMSCs), murine myoblasts (C2C12), and murine embryonic fibroblasts (NIH-3T3) in a freeze-dried state. Freeze-drying reduces the specimen to the nonvolatile components and yields a high signal/noise. Therefore, this state is well suited to develop the technique before translation to the physiologically more relevant aqueous state. Results presented here have been obtained using two different synchrotron beamlines: nano-SAXS was performed at the coherent nanofocus endstation Göttingen Instrument for Nano-Imaging with X-Rays at the P10 beamline at the PETRA III storage ring at DESY in Hamburg, Germany, and micro-SAXS was performed at the cSAXS beamline at the Swiss Light Source at the Paul Scherrer Institut in Villigen, Switzerland. Our main hypothesis is that the electron density variations in naive stem cells are comparatively low, indicative of a weak structural organization, while the differentiated cells may exhibit stronger Fourier components, as expected for cell types that exhibit higher structural order of their cytoskeleton, e.g., myoblasts.

**Figure 1:**
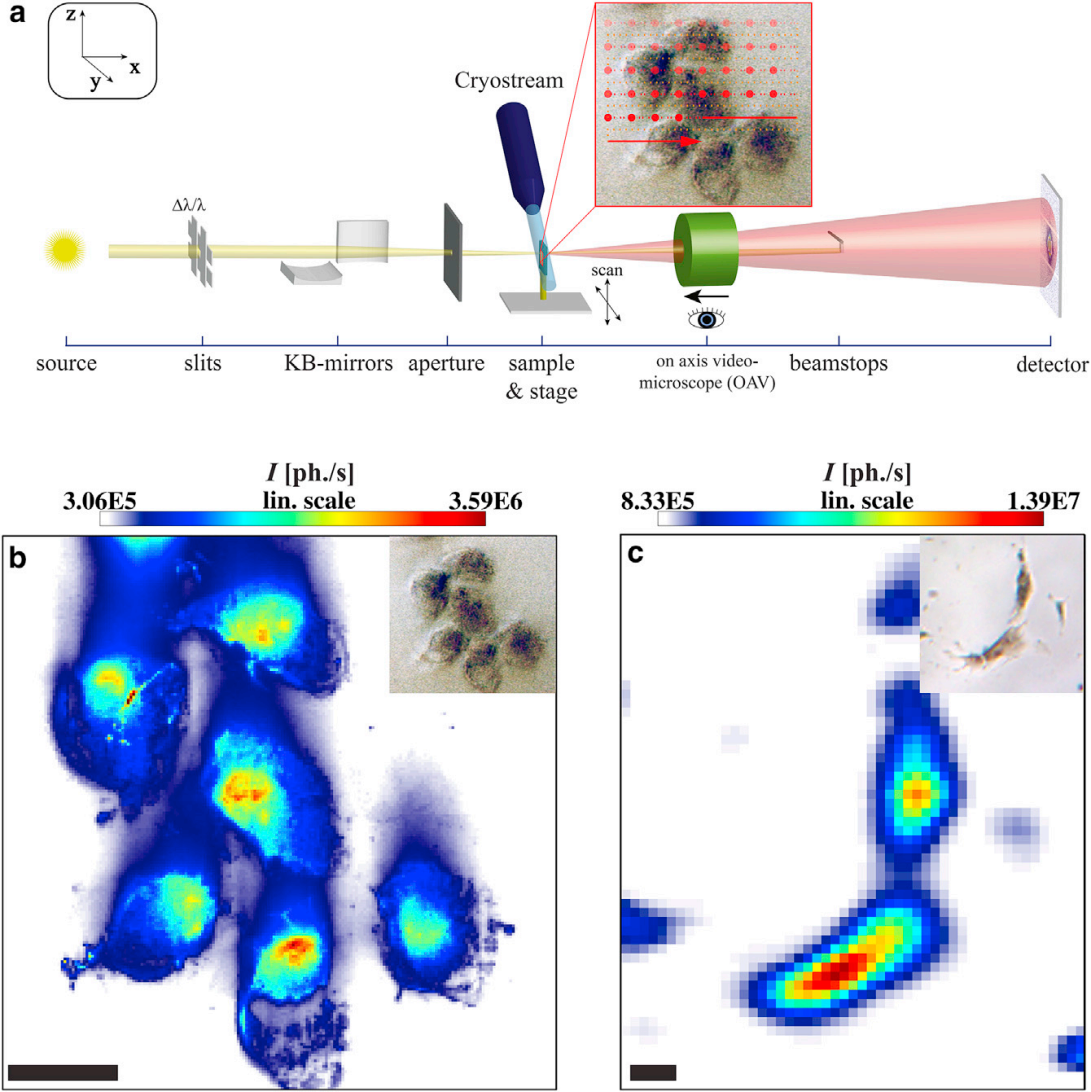
(*a*) Schematic of major optical components of a SAXS experiment with a nanofocused beam. (*b*) X-ray dark field image of lyophilized murine fibroblasts on glass, recorded with the nano-SAXS setup. Micrograph of the sample from the on-axis microscope, right before scanning (*inset*). Scale bar, 20 *μ*m. (*c*) X-ray dark field image of lyophilized naive hMSCs on Si_3_N_4_-membranes recorded with a micro-SAXS setup and micrograph of the sample before scanning (*inset*). Scale bar, 40 *μ*m. To see this figure in color, go online.

Fig. 1 *a* illustrates the basic concept of SAXS recordings with focused beams: an undulator-generated x-ray beam is monochromatized and focused. While the primary beam is blocked, scattered photons are recorded in the far field using a two-dimensional (2D) single photon counting detector. After defining a suitable region of interest via an on-axis video (OAV) microscope, the sample is scanned using a piezo stage. The result is an array of diffraction images, which can be converted to a real space map of different structural observables. In the simplest case, the scattered photons are integrated for each 2D diffraction image, resulting in an x-ray dark field image of the sample $I_{\text{diffraction}}\left(y,z\right)$ as a function of the relative scan positions *y* and *z*. Representative dark field maps are shown in Fig. 1 *b* (freeze-dried murine fibroblasts recorded with the nano-SAXS setup) and in Fig. 1 *c* (freeze-dried naive hMSCs recorded with the micro-SAXS setup). Samples are aligned and monitored during the x-ray scans by the OAV; see Fig. 1 b (*upper right*). When comparing the two datasets, the higher real space resolution of the nano-SAXS scan (0.5 *μ*m step size) compared to the micro-SAXS dataset (8 *μ*m step size) becomes immediately apparent. Although the beamsize limits the real space resolution on the one hand, one must keep in mind that relaxed focusing conditions, resulting in an almost parallel propagating beam, may enable cleaner and more highly resolved diffraction patterns, as well as lower local dose on the other hand.

This article is organized as follows: after this Introduction and the setup conditions described in Materials and Methods, a method to reduce radiation damage in cells by a cryogenic sample protection is presented in Cryogenic Protection of Samples. Nano- and micro-SAXS results on freeze-dried samples are shown in Freeze-Dried Samples and Structure Factor with an emphasis on anisotropies in the diffraction patterns. Analysis of Anisotropy in the Diffraction Patterns then describes an automated empirical analysis of the diffraction patterns based on principal component analysis (PCA). A comparison of the structural parameters derived for all cell lines is given in Comparison of Different Cell Types, pointing at pronounced cell type-specific differences, before the article closes with a brief summary and Conclusion.

## Materials and Methods

### Sample preparation

#### Substrates

hMSC, mi-hMSC, NIH-3T3, and C2C12 were prepared on Si_3_N_4_-ultrathin membranes (Silson, Warwickshire, UK) of 1 *μ*m-thickness or glass coverslips of ≈100 *μ*m-thickness. Membranes and glass coverslips were plasma-cleaned before preparation. A drop of medium was pipetted on top of the substrate at room temperature, followed by a second drop after more than 5 min. The substrate was rinsed with 2 mL of Dulbecco’s phosphate-buffered saline (DPBS). After detachment and washing of the cell-stock, a droplet of the cell suspension of typically 20 *μ*L (100–200 cells/*μ*L) was brought onto the partially wet and coated substrate. Cells settled for ∼10 min at room temperature. This step was carried out two to three times. After settlement, the samples were immersed in 2 mL of prewarmed nutrition medium, and incubated at 37°C/5%CO_2_ for up to 5 days. For C2C12-cells in a commercially available chamber cell, suspension (40 cells/*μ*L) was brought into a collagen IV-coated *μ*-slide with a channel height of 200 *μ*m. Sample was fixed the next day. The self-assembled glass chamber was formed using a second coverslip. A hole-punched parafilm segment was placed on top. A droplet of DPBS was placed into the hole in the center. Next, the cell-containing glass coverslip was placed upside-down, and the parafilm was locally heated forming a tight chamber. The chamber was finally sealed using nail polish.

#### Cells

See Section S10.2 in the Supporting Material.

#### Sample fixation

##### Cryofixation

The samples were imaged by phase contrast microscopy right before plunging. The substrates were blotted manually, plunged using the commercially available grid plunger model EM GP (Leica Microsystems, Wetzlar, Germany), and stored in liquid nitrogen thereafter. Lyophilization was done in a home-built freeze-drier for 3 days and all samples were transferred to silica gel-filled desiccators where they remain until shortly before the x-ray measurements.

##### Chemical fixation

Samples were fixed using prewarmed 9% formaldehyde in DPBS. All samples were washed three times and stored in DPBS in the refrigerator.

### Instrumentation

#### Nanofocus setup

Nano-SAXS data were recorded at the P10-beamline of DESY’s PETRAIII storage ring, using the Göttingen Instrument for Nano-Imaging with X-Rays (24). Freeze-dried and chemically fixed samples in glass chambers were recorded at a photon energy of *E*_photon_ = 7.9 keV (P10/first run). The beam was focused by a pair of Kirkpatrick-Baez (KB) mirrors to a size of ≈250 × 320 nm (vertical × horizontal, full width at half-maximum (FWHM)) as measured by translation of a waveguide. Two soft-edge apertures were placed in front of the sample to block parasitic x-rays from the mirrors (25). The focused photon flux measured at the detector was *I*_0_ = 1.29 × 10^11^ photon/s. The beam could be attenuated using a set of aluminum foils. The sample was mounted on a motorized stage and placed in the focus at ≈20 cm downstream from the KB-mirrors. An OAV microscope with 10–30 × optical zoom, a working distance of 50 mm, and LED illumination was used for alignment and to define suitable scan regions. Optionally, cryogenic conditions could be applied to the sample via a cryogenic nitrogen gas stream (Oxford Instruments, Abingdon, Oxfordshire, UK). The diffraction patterns for each scan point were recorded by the single photon counting pixel detector Pilatus 300k (Dectris, Baden, Switzerland), positioned ≈5.1 m behind the focal plane. To reduce absorption, the beam was guided through an evacuated tube. The primary beam was blocked by multiple semitransparent beamstops. Scans were performed in a discrete or continuous mode with a step size of typically Δ_real_ = 0.5–1 *μ*m, defining the real space resolution. In the discrete scanning mode, the beam was blocked by a fast shutter when moving the sample, while in continuous mode the beam shutter remained open when scanning along the fast axis, i.e., along the *y* direction. Recordings of hydrated cells in commercial cell culture chambers were performed at *E*_photon_ = 13.8 keV (P10/second run) with a maximum flux of *I*_0_ = 1.38 × 10^11^ photons/s measured at the detector and a focus size of ≈180 × 370 nm (vertical × horizontal, FWHM) as measured by translation of a waveguide. A set of molybdenum foils was used for attenuation, all other parameters were comparable to the P10/first run.

#### Microfocus setup

Micro-SAXS data were recorded at the cSAXS-beamline of the Swiss Light Source. The x-ray beam was focused by a Si(111) crystal monochromator in the horizontal and a Rh-coated mirror in the vertical direction resulting in a photon energy of *E*_photon_ = 8.7 keV and a beamsize of ∼33 × 54 *μ*m (vertical × horizontal, FWHM) at the sample position. The primary flux measured at the detector was *I*_0_ = 1.44 × 10^11^ photon/s. The sample was positioned in the focal plane at ≈5 m downstream from the mirrors. Multiple step motors and a hexapod enabled the translation of the sample; the step size varied from typically 5–10 *μ*m. A 10× on-axis optical microscope was used for alignment and surveillance of the sample. The shape and size of the beam at the sample position were determined by a scintillator based x-ray-microscope. An evacuated tube of 7 m length spans the distance from the sample to the single photon counting pixel detector Pilatus 2M (26).

For an overview of the fundamental setup parameters, see Table 1.

## Results

### Cryogenic protection of samples

**Figure 2:**
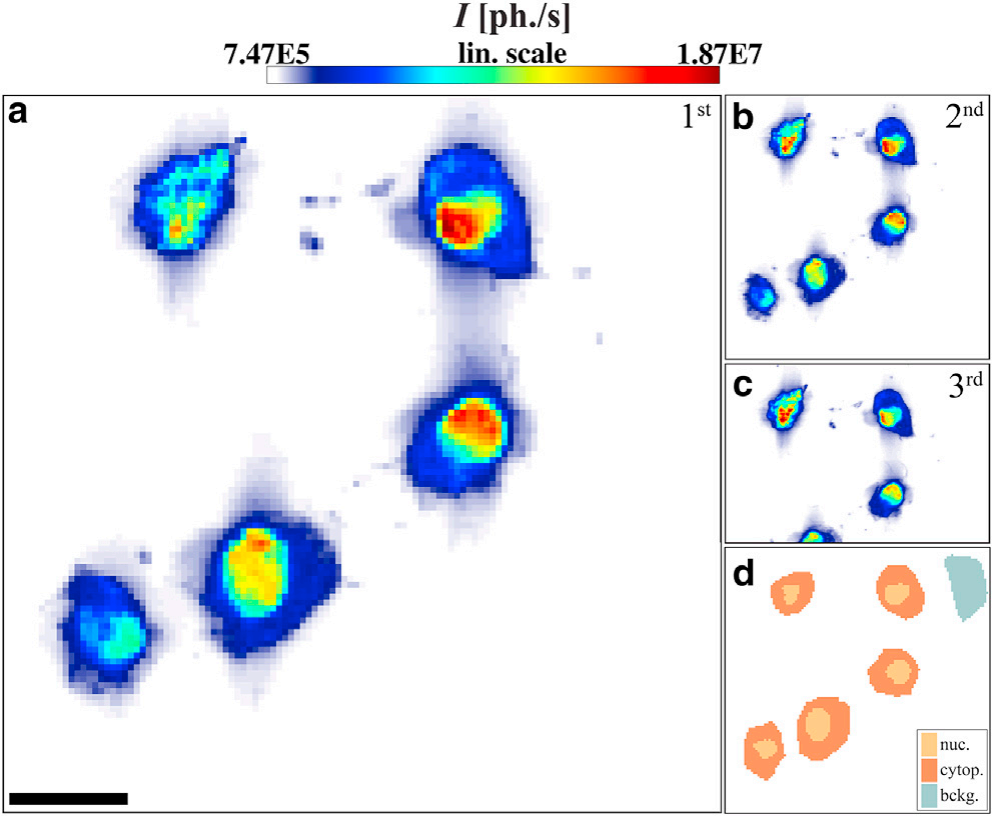
Images of five freeze-dried murine fibroblasts. (*a–c*) X-ray dark field maps of lyophilized murine fibroblasts on a Si_3_N_4_-membrane recorded with a nanofocused beam with samples kept in a cryogenic gas stream to suppress radiation damage. Three subsequent scans (*a*, *b*, and *c*) are recorded, the first two with a step size of Δ_*y*,*z*_ = 1 *μ*m, the last with Δ_*y*,*z*_ = 0.5 *μ*m. Scale bar, 20 *μ*m. (*d*) Dark field mask, defining nucleic and cytoplasmic regions for further quantification. To see this figure in color, go online.

Cells within the scan area of the focused beam suffer from high radiation dose, causing structural damage. The radiation dose applied in every scan point is given by

(1) $$D=\frac{I_{0}\times \tau \times E_{\text{photon}}}{l_{\text{attenuation}}\times \rho \times \Delta_{y}\times \Delta_{z}},$$

with the intensity *I*_0_ in photons/s, dwell time *τ* in s, photon energy $E_{\text{photon}}=7.9\;\text{keV}$, the focal spot size Δ_*y*_ and Δ_*z*_ (FWHM), and the attenuation length $l_{\text{attenuation}}=7.2\times 10^{-4}\;\text{m}$ evaluated for a hypothetical model protein H_50_ C_30_ N_9_ O_10_ S with a given density *ρ* = 1.35 g/cm^3^ (27). Radiation damage at high dose can, for example, manifest itself by changes in the radial intensity profile $I\left(q_{\text{r}}\right)$ and an overall reduction in the scattering intensity in subsequent scans on the same area. One well-known approach to minimize radiation damage is to use cryogenic sample conditions. According to Meisburger et al. (28), cryo-cooled proteins and nucleic acids can withstand doses at least two orders-of-magnitude larger than samples at room temperature. To verify that radiation damage did not affect the recorded signals in a significant way under cryogenic conditions, we performed a scan series on freeze-dried murine fibroblasts applying a dose of *D* ≈ 2.1 × 10^8^ Gy in each of three successive scans, while keeping the sample in a cryogenic gas stream (Oxford Instruments). As shown in Fig. 2, *a–c*, the contrast images are indeed perfectly consistent and without any significant structural alterations. For the x-ray dark field value shown in each of the image pixels, the diffraction signal was integrated over the detector, excluding primary beam {pb} and setup-related parasitic scattering {ps}

(2) $$I_{\text{diffraction}}\left(y,z\right)=\int_{R^{2}\setminus \left[\left\{\text{pb}\right\}\cup \left\{\text{ps}\right\}\right]}I\left(y,z,q_{y},q_{z}\right)\text{d}q_{y}\;\text{d}q_{z},$$

with $q_{y}$ and $q_{z}$ components of the scattering vector in the *y*-*z* plane.

To further quantify possible structural alterations, we have evaluated the radial intensity profiles of the scans and averaged over all pixels in a certain cellular region, as defined by a software mask separating cytoplasms and nuclei (Fig. 2 d). After calculating the corresponding averaged 2D diffraction image, an azimuthal average is determined to yield a one-dimensional radial intensity profile $I\left(q_{\text{r}}\right)$. Profiles are fitted to a power-law decay, after subtraction of a constant background signal (22)

(3) $$I\left(q_{r}\right)=a\times q_{r}^{b}+c\;,$$

with the scattering vector component $q_{r}=\sqrt{q_{y}^{2}+q_{z}^{2}}$ perpendicular to the primary beam on the detector plane. Results of the scan series are listed in Table 2: one five-cell arrangement recorded by three subsequent scans and one three-cell arrangement recorded by two subsequent scans (see Section S10.3 in the Supporting Material). They reveal only slight deviations in the exponent *b* (see Table 2), which are not significant in view of the statistical and systematic errors of the procedure. In particular, we do not observe a steepening in the power law decay, as observed in Priebe et al. (22) for samples suffering from beam-induced damage. We conclude that cryoprotection preserves freeze-dried cells sufficiently for nano-SAXS scans.

### Freeze-dried samples and structure factor

**Figure 3:**
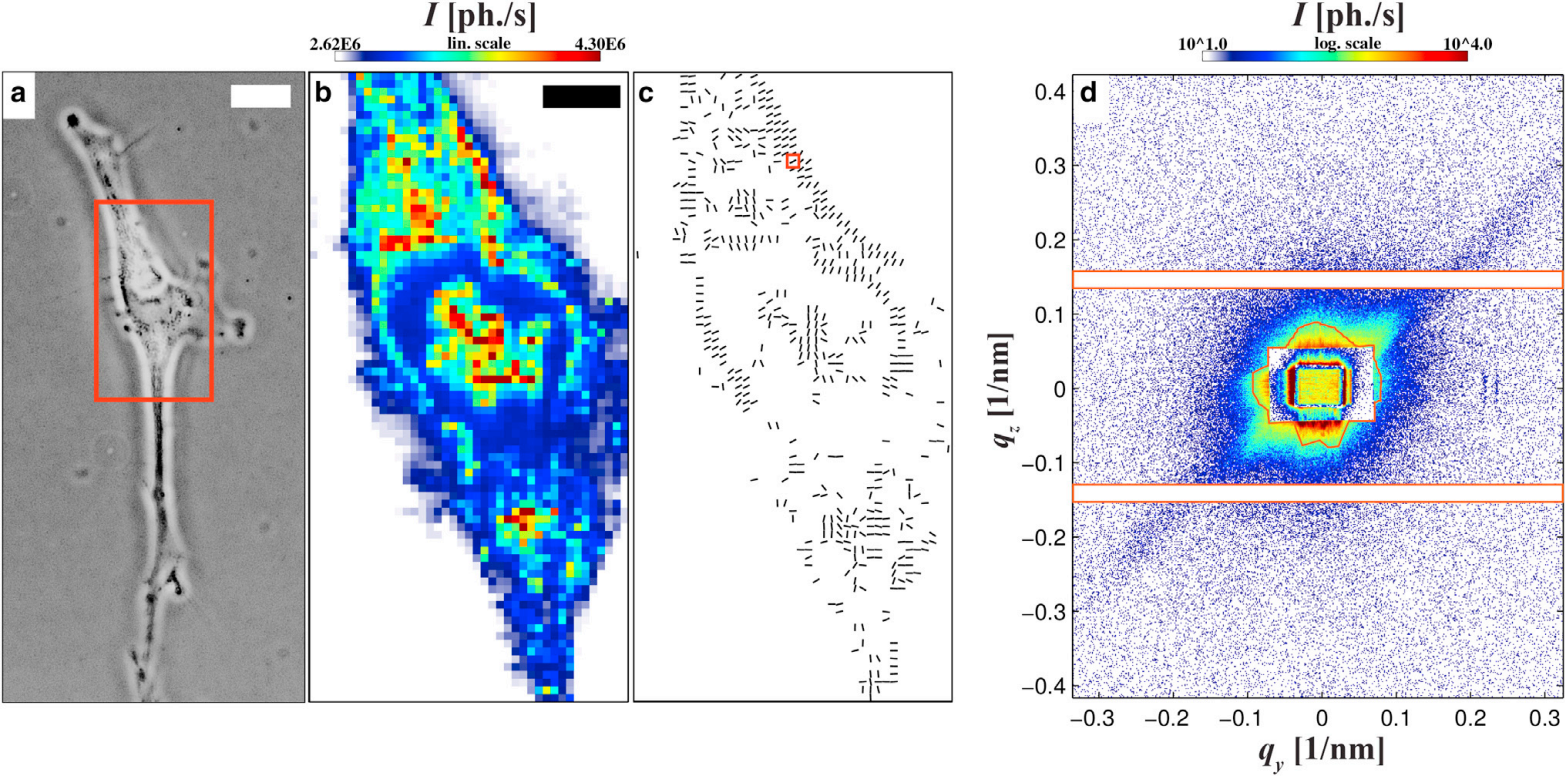
(*a*) A 40× phase contrast image of a mi-hMSC recorded right before cryo plunging. The red frame shows the approximated scan region. Scale bar, 20 *μ*m. (*b*) X-ray dark field map recorded with the nano-SAXS setup. Scale bar, 5 *μ*m. (*c*) Anisotropic diffraction, evaluated as in Priebe et al. (22), visualizing the anisotropy in terms of the direction of the diffraction peak. (*d*) Single diffraction image at the position marked in (*c*) also showing rectangular shadows of the semitransparent beamstops in the center. The detector mask is indicated as orange frames. To see this figure in color, go online.

We have performed multiple scans on freeze-dried samples using the nano- and micro-SAXS setup. We first show nano- and then micro-SAXS results, illustrating typical data that can be obtained in scanning SAXS experiments on cells, and simple approaches to quantify the radial intensity decay. Even if not yet analyzed with a model, the quality of the diffraction data underlines the potential of the label-free imaging technique to reach high resolution even in weakly scattering cells. Fig. 3 shows a representative nano-SAXS dataset of a freeze-dried mi-hMSC mounted on a Si_3_N_4_-membrane, recorded with a step size of Δ_*y*,*z*_ = 0.5 *μ*m. A phase contrast visible light micrograph of the cell (40×) before cryo-plunging is shown in Fig. 3 *a*. The region indicated by the red rectangle corresponds to the approximated scanning region with its nano-SAXS dark field map shown in Fig. 3 *b*, revealing position and shape of the cell with its nucleus, which can be distinguished from the cell’s periphery, i.e., the cytoplasm, by a change in scattering cross section. To identify anisotropies in the diffraction patterns, the so-called streakfinder algorithm (22) is applied, and the orientation is plotted for all pixels with anisotropy exceeding a selected threshold (parameters *S* > 0.09 as defined in Priebe et al. (22)). Regions of consistent orientation can be recognized: neighboring pixels reveal a similar orientation while the overall orientation coincides with the expectation that the stress fiber alignment is predominantly along the extensions of adherent cells (4). Pronounced anisotropies occur close to the plasma membrane; see single diffraction image in Fig. 3 *d*, located at the position marked in Fig. 3 *c*.

**Figure 4:**
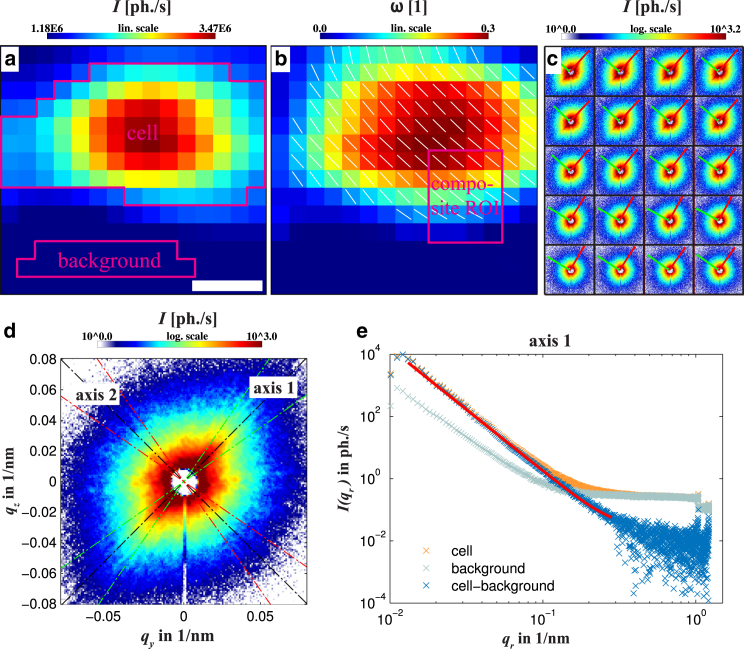
(*a*) X-ray dark field image of a freeze-dried murine myoblast recorded with the micro-SAXS setup. Scale bar, 20 *μ*m. (*b*) PCA result: White lines indicate the principal orientation axis. The anisotropy parameter *ω* (see Eq. 9) is color-coded. (*c*) Composite of the region marked in (*b*). Each diffraction pattern is cropped to a region of *q*_*r*_ < 0.11 nm^−1^. The corresponding eigenvectors are scaled by their relative standard deviation and depicted as red and green arrows (for further explanations, see Analysis of Anisotropy in the Diffraction Patterns). (*d*) Average diffraction signal of the cell. The region taken into account is marked in (*a*). The two axes resulting from PCA indicate the principal directions of anisotropy, the directions of lowest and highest variance. (*e*) Averaged radial intensity profile of a segment ±10° around axis 1. Data are then fitted by a power law function following Eq. 4, resulting in *b* = −3.95 (axis 1) and *b* = −3.74 (axis 2), not shown. To see this figure in color, go online.

Nanodiffraction allows us to investigate highly localized structures in the cell, probed in each diffraction point. However, focusing is accompanied by diffraction broadening of the primary beam behind the sample, decreasing the angular resolution Δ*q* in the detection plane. We therefore complement the nano-SAXS data by micro-SAXS recordings with lower resolution in real space, but higher resolution in reciprocal space, and moreover less parasitic scattering, because the focusing is relaxed. This is implemented at the cSAXS beamline, providing an isotropic and clean direct beam signal in the far field without the typical artifacts of KB focusing. Fig. 4 shows one example of a scan performed on freeze-dried murine myoblasts on a Si_3_N_4_-window. The x-ray dark field image reveals high scattering intensity in the center of the cell (Fig. 4 *a*). The beam size, which is in the same order of the cell size, blurs the real-space image and the contour of the nucleus and the cell membrane are not clearly seen. The clean diffraction signal enables PCA (see Fig. 4 *b*). Local anisotropies can be quantified by definition of an anisotropy parameter *ω* (color-coded), as detailed in Analysis of Anisotropy in the Diffraction Patterns. Orientations of the spatial structures are perpendicular to the scattering signal and are depicted as white lines. In agreement with the composite image (Fig. 4 *c*), pronounced and consistent anisotropy is found throughout the entire cell, with a maximum in its center. To obtain additional structural information, an average cell diffraction signal is computed, onto which PCA is applied (Fig. 4 *d*). The PCA yields the two principal (orthogonal) axes k∈[1,2] with corresponding standard deviation of the photons’ momentum transfer $\sigma_{k}$ as a model-free parameter for the scattering distribution, here $\sigma_{1}=1.9\times 10^{-2}$ nm^−1^ and $\sigma_{2}=1.3\times 10^{-2}$ nm^−1^, respectively. By definition, $\sigma_{i}$ values are calculated in units of nm^−1^ corresponding to real space length of $d_{1}=$ 169 nm and $d_{2}=$ 238 nm, with $d_{k}=\pi /\sigma_{k}$. Next, we analyze the decrements of the radial intensity profile $I\left(q_{\text{r}}\right)$. To this end, two sectors are defined ranging $\pm 10^{\circ }$ around each axis with orientation $\gamma_{k}$. The signal is then transformed into polar coordinates, yielding the radial intensity distributions $I\left(q_{\text{r}}\right){|}_{\gamma_{k}\pm 10^{\circ }}$ along the principal direction. The signal is fitted to a power law decay

(4) $$I\left(q_{\text{r}}\right)=a\times q_{\text{r}}^{b}+c.$$

Fit parameters are listed in Table 3.

### Analysis of anisotropy in the diffraction patterns

To quantify the anisotropy of the SAXS patterns, we apply PCA to the diffraction patterns. PCA diagonalizes the covariance matrix

(5) $$C=\left(\begin{array}{cc} \text{var}\left(q_{y}\right) & \text{cov}\left(q_{y},q_{z}\right)\\ \text{cov}\left(q_{z},q_{y}\right) & \text{var}\left(q_{z}\right) \end{array}\right)$$

with

(6) $$\text{cov}\left(q_{i},q_{j}\right)=\frac{\sum_{m,n}I\left(m,n\right)\times \left[q_{i}\left(m,n\right)-\langle q_{i}\rangle \right]\times \left[q_{j}\left(m,n\right)-\langle q_{j}\rangle \right]}{\sum_{m,n}I\left(m,n\right)}$$

and the variances $\text{var}\left(q_{i}\right)=\text{cov}\left(q_{i},q_{i}\right)$, i=y,z as elements. I(m,n) denotes the photon counts in pixel m,n∈N on the (masked) detector. The expectation value $\langle q_{i}\rangle$ for a single photon is given by

(7) $$\langle q_{i}\rangle =\frac{\sum_{m,n}I\left(m,n\right)\times q_{i}\left(m,n\right)}{\sum_{m,n}I\left(m,n\right)}\;,$$

which in most experiments is the center of the primary beam. Solving the corresponding eigenvalue problem

(8) $$C\times {\vec{b}}_{k}=\lambda_{k}\times {\vec{b}}_{k}$$

yields two eigenvectors ${\vec{b}}_{k}$, k∈[1,2], which form a new orthogonal basis. The eigenvalues $\lambda_{k}=\sigma_{k}^{2}$ correspond to the variance in either direction. Both eigenvectors are scaled to unity $\left({\vec{e}}_{k}={\vec{b}}_{k}/\left\|{\vec{b}}_{k}\right\|\right)$ and then sorted: the vector related to the largest eigenvalue describes the “line of best fit” (29, 30), and is in the following denoted as the principal axis ${\vec{e}}_{\text{pa}}$, implying an orientation angle $\gamma_{\text{pa}}$. Because the cellular structure causing the signal is directed perpendicular to the diffraction pattern, the structure orientation angle $\theta_{\text{pa}}$ is defined by $\theta_{\text{pa}}=\gamma_{\text{pa}}+90^{\circ }$. The anisotropy of the diffraction pattern is then defined locally for each scan point by the anisotropy parameter

(9) $$\omega =\frac{\left|\lambda_{1}-\lambda_{2}\right|}{\lambda_{1}+\lambda_{2}}.$$

This results in an anisotropy map ω(y,z), which can be plotted along with the map of the orientation angle $\theta_{\text{pa}}\left(y,z\right)$, depicted as a white line, e.g., in Fig. 4 *b*.

**Figure 5:**
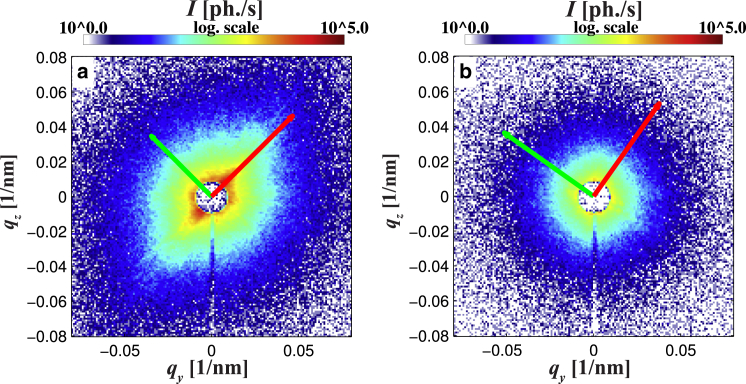
Examples for (*a*) anisotropic and (*b*) isotropic diffraction patterns are shown along with the resulting principal axis of the PCA. Vectors are rescaled by the standard deviation *σ*_*k*_(*y*,*z*) as denoted in Eq. 10. For presentation purposes, a common scaling factor is introduced for the orientation vectors, keeping the aspect ratio unaffected. To see this figure in color, go online.

PCA gives reliable results on SAXS patterns, as long as the empty beam is isotropic and the evaluated area is not interrupted by intermodular gaps of the detector. We thus restrict the evaluation of the diffraction signals to the inner module to avoid all gaps resulting in diffraction images as shown in Fig. 4 *c*. Before analysis, a ring-shape mask is applied setting values around the beamstop to zero. The eigenvectors are rescaled by the standard deviation $\sigma_{k}\left(y,z\right)=\sqrt{\lambda_{k}\left(y,z\right)}$

(10) $${\vec{a}}_{k}\left(y,z\right)=\sigma_{k}\left(y,z\right)\times {\vec{e}}_{k}\left(y,z\right),$$

illustrating the character of a signal: diffraction images with a pronounced anisotropic signal result in a relatively large aspect ratio $A_{\text{r}}\left(y,z\right)=\left|{\vec{a}}_{\text{pa}}\left(y,z\right)\right|/\left|{\vec{a}}_{k\neq \text{pa}}\left(y,z\right)\right|$ (Fig. 5 *a*). Contrarily, for isotropic signals, $A_{r}\left(y,z\right)≃1$ (Fig. 5 *b*). Note that, by definition, ${\vec{a}}_{k}\left(y,z\right)$ values are calculated in units of nm^−1^, supporting the representation of these vectors in reciprocal space.

Next, we consider cellular maps of entire cells and characterize them by defining global parameters to address cell type-specific structural differences. First, we measure orientational variations by calculating the 2D nematic order parameter *s*. The value *s* quantifies the variation of the principal axes’ orientation within a cell. Following Liu et al. (31), we define the second rank tensor *Q*

(11) $$Q_{\alpha ,\beta }=\frac{1}{N_{\text{ROI}}}\sum_{y,z{|}_{\text{ROI}}}\left(2\;e_{\text{pa},\alpha }\left(y,z\right)e_{\text{pa},\beta }\left(y,z\right)-\delta_{\alpha ,\beta }\right),$$

with *α*,*β* = 1,2, the total number of diffraction patterns $N_{\text{ROI}}$ within the region of interest and the principal axis defined by

(12) $${\vec{e}}_{\text{pa}}\left(y,z\right)=\left(\begin{array}{c} \text{cos}\left(\theta_{\text{pa}}\left(y,z\right)\right)\\ \text{sin}\left(\theta_{\text{pa}}\left(y,z\right)\right) \end{array}\right)\;.$$

**Figure 6:**
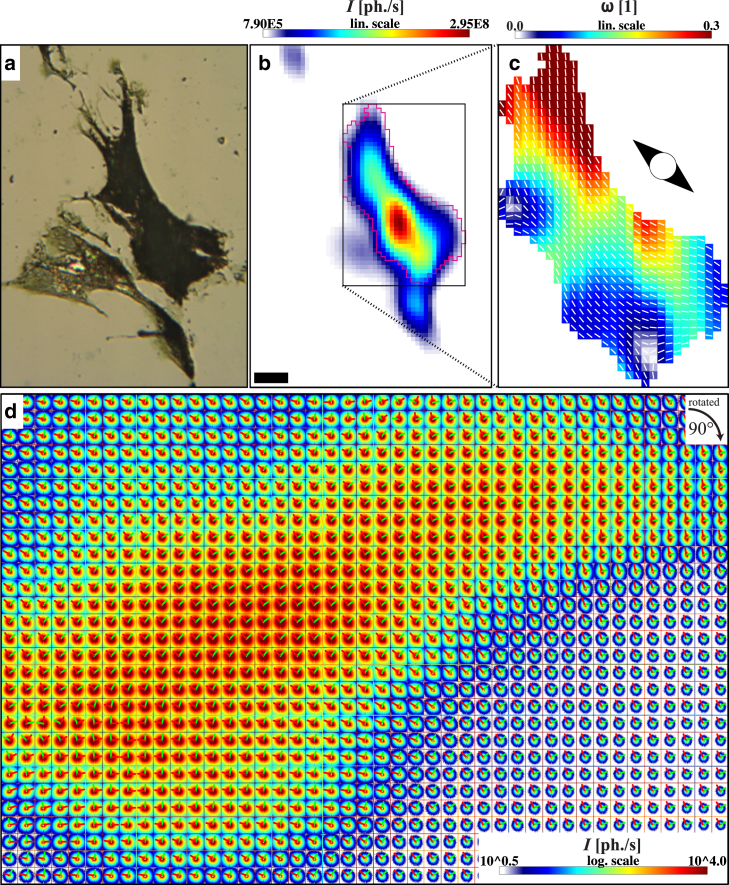
(*a*) OAV-image of lyophilized muscle-induced hMSCs before recording. (*b*) X-ray dark field scan recorded with the micro-SAXS setup. Scale bar, 40 *μ*m. (*c*) PCA of the region marked in (*b*). All contributions outside this area are set to zero. (*d*) Composite image of the scan area shown in (*c*), dataset rotated clockwise by 90°. By PCA, two basis vectors are computed for every diffraction image. To see this figure in color, go online.

Insertion of Eq. 12 in Eq. 11 and solving the eigenvalue problem of *Q* then leads to an expression for the order parameter *s*, being the positive eigenvalue

(13) $$s=\frac{1}{N_{\text{ROI}}}\sqrt{{\left(\sum_{y,z{|}_{\text{ROI}}}\text{sin}\left(2\;\theta_{\text{pa}}\left(y,z\right)\right)\right)}^{2}+{\left(\sum_{y,z{|}_{\text{ROI}}}\text{cos}\left(2\;\theta_{\text{pa}}\left(y,z\right)\right)\right)}^{2}}$$

(for details, see Section S10.5 in the Supporting Material). The corresponding eigenvector is the director of the evaluated area; see the black arrow in Fig. 6 *c*. In addition, an overall anisotropic scattering strength of the cell can be quantified by averaging the local anisotropy parameters ω(y,z)

(14) $$\Omega =\frac{1}{N_{\text{ROI}}}\sum_{y,z{|}_{\text{ROI}}}\omega \left(y,z\right).$$

Fig. 6 shows a freeze-dried mi-hMSC, along with Fig. 6 *a*, the corresponding OAV-image and Fig. 6 *b*, the x-ray dark field map. A region of interest as marked in pink is evaluated by PCA and shown in Fig. 6 *c*; all contributions outside this region are set to zero. PCA results can then be depicted in a composite image, again showing the two rescaled eigenvectors ${\vec{a}}_{k}\left(y,z\right)$ at each scan position in Fig. 6 *d*. This dataset leads to values of s=0.65 and Ω=0.15. Further examples are shown in the Supporting Material (Section S10.5 in the Supporting Material).

### Comparison of different cell types

**Figure 7:**
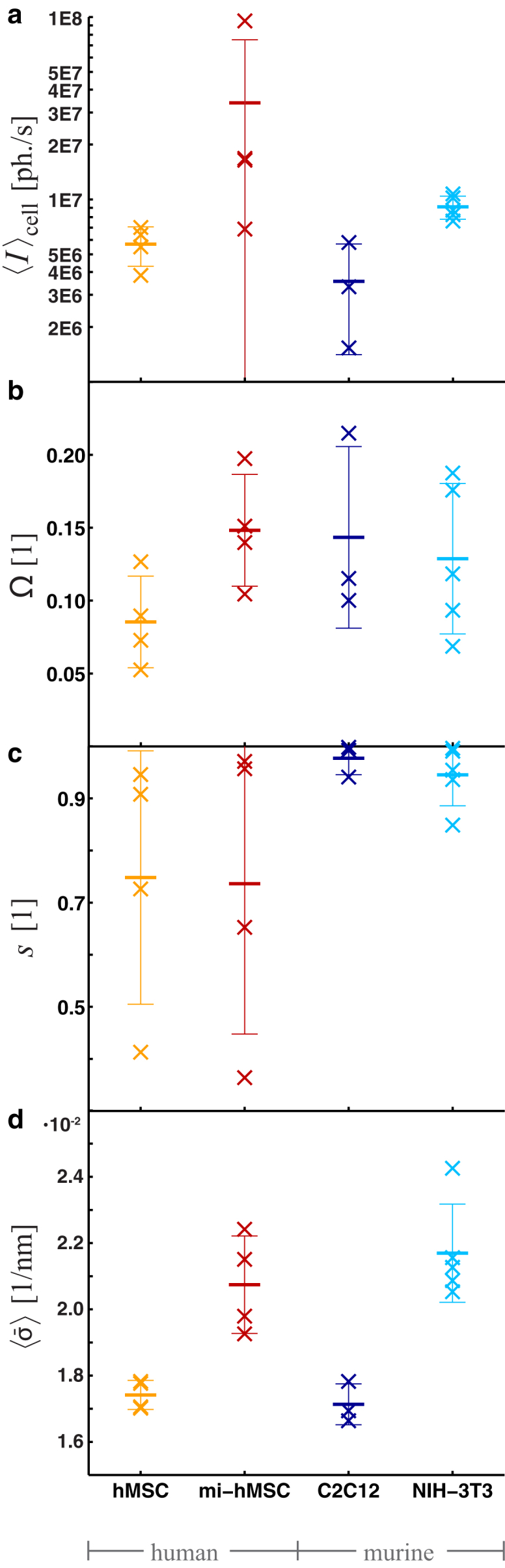
Statistical evaluation of the mean (*a*) scattering intensity, (*b*) anisotropy parameter Ω (Eq. 14), (*c*) 2D nematic order parameter *s* (Eq. 13), and (*d*) averaged standard deviation of diffraction patterns $\bar{\sigma }$ for different cell types. To see this figure in color, go online.

To compare different cell types in view of the structural observables defined above, the results of multiple scans performed on freeze-dried naive and mi-hMSCs, as well as murine myo- (C2C12) and fibroblasts (NIH-3T3) are compiled in this section (Fig. 7). For analysis, the x-ray dark field image of every scan was masked separating the diffraction data into areas of relatively strong and weak scattering signals. Comparison of dark field and OAV-image then leads to an adjusted mask, defining an adequate region to calculate these observables for single cells as detailed in Section S10.5 in the Supporting Material. Fig. 7 *a* shows the results for the mean scattering intensity ${\langle I\rangle }_{\text{cell}}$ for single cells and different cell types:

(15) $${\langle I\rangle }_{\text{cell}}=\frac{1}{N_{\text{ROI}}}\sum_{y,z{|}_{\text{ROI}}}I_{\text{diffraction}}\left(y,z\right).$$

The mean for each cell type is depicted as bars (and the standard deviation as error bars) showing an increase in scattering intensity from naive to mi-hMCSs and from murine myo- to fibroblasts.

Because contributions to ${\langle I\rangle }_{\text{cell}}$ can originate from both isotropic and anisotropic portions of the signal, data are further analyzed in this respect by the determination of Ω for every cell following Eq. 14 (Fig. 7 *b*). When comparing naive hMSCs to all other cell types, a shift in data is apparent, supporting our hypothesis of the former being rather unstructured.

Addressing orientational variations, we then calculate the 2D nematic order parameter *s*, leading to relatively high values for all murine cells (Fig. 7 *c*). In fact, regarding the local orientation angles θ(y,z) of murine cells, a strict overall orientation is apparent (see also Section S10.5 in the Supporting Material).

Furthermore, we quantify the radial intensity profile $I\left(q_{\text{r}}\right)$ by determining the mean standard deviation ${\langle \bar{\sigma }\rangle }_{\text{cell}}$, which is a convenient and straightforward measure of a representative scale of Fourier components, independent of any particular model or fitting ansatz with

(16) $${\langle \bar{\sigma }\rangle }_{\text{cell}}=\frac{\sum_{{y,z|}_{\text{ROI}}}\bar{\sigma }\left(y,z\right)}{N_{\text{ROI}}}=\frac{\sum_{y,z{|}_{\text{ROI}}}\sqrt{\frac{\lambda_{1}\left(y,z\right)+\lambda_{2}\left(y,z\right)}{2}}}{N_{\text{ROI}}}$$

(Fig. 7 *d*). Relatively large values for ${\langle \bar{\sigma }\rangle }_{\text{cell}}$ are obtained in case of mi-hMSCs and murine fibroblasts implying significant scattering for high *q* values caused by small, ordered structures within the cells.

## Discussion

Understanding cellular processes in general, and stem cell differentiation in particular, requires advanced biophysical methods to unravel the evolution of subcellular structures. Here we apply scanning small-angle x-ray scattering with focused beams to biological cells to probe the native electron density distribution. While the full potential of these methods will probably only unfold once suitable modeling of the local and highly anisotropic diffraction patterns becomes available, we here adopt a strategy of automatized empirical analysis of the diffraction data. In particular, we show that PCA is suited to track down local orientation angles and the degree of anisotropy under the precondition of a sufficiently isotropic direct beam. From PCA results, a global nematic order parameter can be derived in a straightforward manner. In particular, we have introduced four structural parameters, which can be computed from the scanning SAXS data in a model-free approach: the mean scattering intensity ${\langle I\rangle }_{\text{cell}}$; the mean anisotropy parameter Ω; the 2D nematic order parameter *s*; and the mean variance of the momentum transfer $\langle \bar{\sigma }\rangle$, corresponding to typical length scales dominating the diffraction. This approach provides surprising insight into the evolution of local structures and anisotropy of the cytoskeleton. While we cannot trace down the individual molecular components producing the diffraction, we can record and analyze the locally dominating Fourier components up to near-molecular scales with a real-space resolution, which is still high enough to distinguish between different parts of the cell, such as nucleus or cytoplasm. The results support the hypothesis that naive hMSCs are comparatively void of structure, lacking the pronounced Fourier components and anisotropies observed in the diffraction patterns of other cell types. Apart from these first results, which require further work and extension, the primary goal of this work was to further establish cellular micro- and nano-SAXS as a novel label-free imaging technique, enhanced by technical developments including optical setups (recent publications demonstrated beam focusing in the nanometer-range (32, 33)), sample environments such as highly transmissive chambers (Silson, Northampton, UK), and the analysis tools described here. Altogether, diffraction data from biological cells is recorded at a signal/noise that was believed to be impossible just a few years ago. Importantly, we showed that cryogenic conditions can suppress beam damage and preserve the structure of the specimen allowing multiple scans or even the combination of different recording methods (e.g., nano-SAXS and ptychography (19, 34) or nano-SAXS and holography (23)) on the same area. At the same time, it becomes clear that hydrated cells are much more challenging, concerning firstly suitable x-ray compatible cell culture chambers, and secondly the achievable signal/noise. To this end, future work needs to address further optimization, in particular in view of suitable window materials, with high transmission and low background in combination with good adhesion probabilities for the cells of interest. Reduction in channel depth, photon energy, and better background subtraction also need to be addressed, as well as possible mechanisms to reduce radiation damage in the room temperature setting, for example by constant flow, free radical scavengers, and measurement protocols.

## Author Contributions

T.S. and F.R. designed research; M.B. prepared samples; M.B. and C.W. established sample preparation workflow; A.D., T.S., and M.B. conceived and implemented the beamline setup; M.B., M.P., T.S., F.R., and M.O. prepared the beamtime and recorded data; M.B. analyzed data; T.S., F.R., M.P., C.W., and M.O. gave constant scientific feedback; and M.B., T.S., M.P., and F.R. wrote the manuscript.

## Figures and Tables

**Table 1:** Setup Parameters of the X-Ray Experiments

Beamline	Beam Diameter		*E* (keV)	*λ* (pm)	*I*_0_ (photons/s)
Horizontal	Vertical
DESY/PETRAIII/P10 (first run)	320 nm	250 nm	7.9	156.9	1.29 × 10^11^
DESY/PETRAIII/P10 (second run)	370 nm	180 nm	13.8	89.8	1.38 × 10^11^
PSI/SLS/cSAXS	54 *μ*m	33 *μ*m	8.7	142.5	1.44 × 10^11^

**Table 2:** Fit Results for Cryoprotected Scan Series

Scan	Region of Interest	Run	*a* ((photons × nm)/s)	*b* (1)	*c* (photons/s)	*R*^2^ (1)
Five cells	cytop-bckg	first	3.2 × 10^−2^	−4.14	1.5 × 10^−1^	0.9998
second	3.3 × 10^−2^	−4.12	1.3 × 10^−1^	0.9998
third	3.8 × 10^−2^	−4.08	1.7 × 10^−1^	0.9998
nuc-bckg	first	1.1 × 10^−1^	−4.29	3.0 × 10^−1^	0.9996
second	8.4 × 10^−2^	−4.31	2.9 × 10^−1^	0.9996
third	6.5 × 10^−2^	−4.28	3.2 × 10^−1^	0.9997
Three cells	cytop-bckg	first	2.5 × 10^−2^	−4.37	1.7 × 10^−1^	0.9997
second	2.2 × 10^−2^	−4.38	1.6 × 10^−1^	0.9997
nuc-bckg	first	1.0 × 10^−1^	−4.34	4.9 × 10^−1^	0.9999
second	7.9 × 10^−2^	−4.35	4.4 × 10^−1^	0.9999

cytop-bckg, cytoplasmic signal minus background signal; nuc-bckg, nucleic signal minus background signal.

**Table 3:** Fit Results following Eq. 4 Applied on Murine Myoblasts Depicted in Fig. 4

Axis	*a* ((photons × nm)/s)	*b* (1)	*c* (photons/s)	*R*^2^ (1)	*σ* (1/nm)	*d* (nm)
1	2.0 × 10^−4^	−3.95	2.7 × 10^−2^	0.97	1.9 × 10^−2^	169
2	1.2 × 10^−4^	−3.74	3.1 × 10^−2^	0.96	1.3 × 10^−2^	238

## Supporting Material

Document S1. Supporting Materials and Methods, Figs. S1–S11, and Table S1

Document S2. Article plus Supporting Material
