# Supplementary material for: X-Ray Micro- and Nanodiffraction Imaging on Human Mesenchymal Stem Cells and Differentiated Cells
Source: Biophys J. 2016 Feb 2;110(3):680–90. doi: 10.1016/j.bpj.2015.12.017 (PMC4744168; doi:10.1016/j.bpj.2015.12.017)
Supplement: Document S1. Supporting Materials and Methods, Figs. S1–S11, and Table S1 [file mmc1.pdf]

Biophysical Journal

Supporting Material

X-Ray Micro- and Nanodiffraction Imaging on Human Mesenchymal Stem Cells and Differentiated Cells

Marten Bernhardt,[1] Marius Priebe,[1] Markus Osterhoff,[1] Carina Wollnik,[2] Ana Diaz,[3] Tim Salditt,[1,*] and Florian Rehfeldt[2,*]

[1]Institut für Röntgenphysik, Georg-August-Universität Göttingen, Göttingen, Germany; [2]Drittes Physikalisches Institut - Biophysik, Georg-August-Universität Göttingen, Göttingen, Germany; and [3]Paul Scherrer Institut, Villigen, Switzerland

*Correspondence: tsaldit@gwdg.de; florian.rehfeldt@phys.uni-goettingen.de

10.1 X-ray Micro- and Nanodiffraction Setup at cSAXS (PSI) & P10 (PETRAIII)

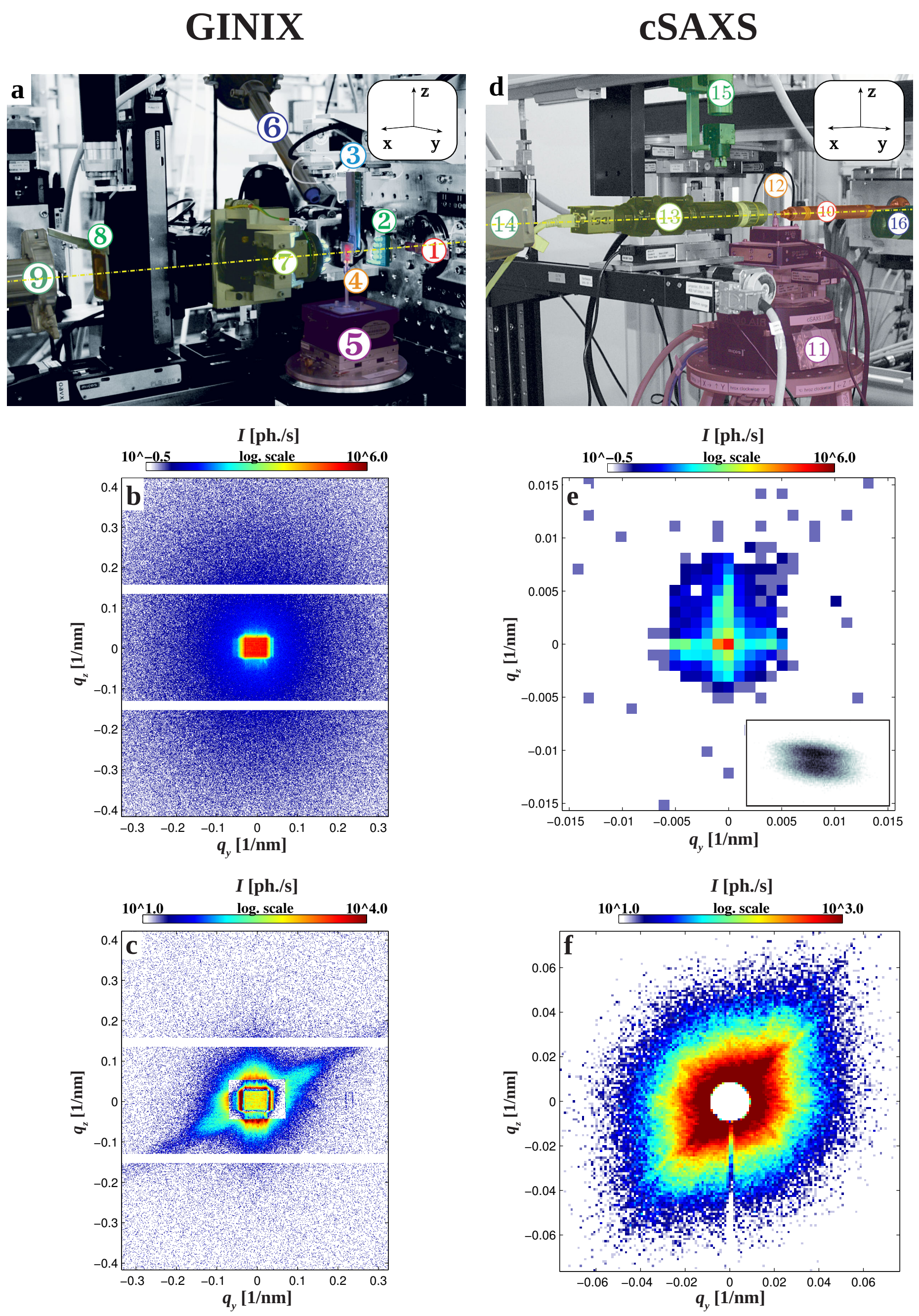

Figure 1: *left:* (*a*) Photograph of the nano-focus setup GINIX (4, 7) together with far field images of (*b*) the direct beam and (*c*) a representative cellular diffraction signal recorded at GINIX. *right:* (*d*) Photograph of the micro-focus setup together with far field images of (*e*) the direct beam and (*f*) a representative cellular diffraction signal recorded at cSAXS (1). The beam profile in focus could be visualized by a scintillator, see *e* (inset).

Fig. 1 *a* shows a photograph of the Göttingen Instrument for Nano-Imaging with x-rays (GINIX) (4) at the P10-beamline of DESY's PETRAIII storage ring: A set

of Kirkpatrick-Baez mirrors (not shown) enabled beam focussing to a size of about 200 nm. The beam left the evacuated tube at (1) impinging the sample (here: a self-assembled glass-chamber, (4)) about 20 cm downstream from the mirrors. Two soft-edge apertures (3) block parasitic scattering. The sample was mounted on a motorized stage (5) enabling sample translation with respect to the beam. An on-axis video-microscope (OAV, Optique Peter, France) (7) with LED illumination (2) was used for orientation on the substrate. Optionally, cryogenic conditions (6) could be applied (Cryostream, Oxford instruments, UK). Diffraction patterns were recorded in ≈ 5.1 m distance by the single photon counting pixelated detector Pilatus 300k (Dectris, Switzerland). An evacuated tube (9) minimized absorption. The primary beam could be blocked by multiple semi-transparent beamstops placed either in front of the tube (8) or inside (not shown). The focused photon flux was $I_0 = 1.29 \cdot 10^{11}$ ph./s as determined by direct beam recordings, see *b*. A representative cellular diffraction image is shown in *c*.
Fig. 1 *d* shows a photograph of the micro-SAXS data at cSAXS-beamline of the Swiss Light Source at the Paul Scherrer Institut in Villigen, Switzerland. The x-ray beam was focused by a Si(111) crystal monochromator in horizontal and a Rh-coated mirror in vertical direction enabling a spotsize of about 40 µm at the sample position. The beam left the vacuum tube through a 4 µm-thick mica-window at (10) impinging the sample (12) about 5 m downstream from the mirrors. Multiple step motors and a hexapod (11) enabled the translation of the sample with respect to the beam. An on-axis optical microscope (13) and a camera (16) were used for orientation on the substrate and the surveillance of the sample. The shape and size of the beam at the sample position was determined by a scintillator based x-ray microscope (15). An evacuated tube (14) of 7 m length spans the distance from the sample to the single photon counting pixelated detector Pilatus 2M (3). For x-ray measurements the on-axis microscope was retracted and the flight tube was approached to the specimen. The focused photon flux was $I_0 = 1.44 \cdot 10^{11}$ ph./s as determined by direct beam recordings with filters, while the setup provides an extraordinarily clean direct beam signal, see *e*. A representative cellular diffraction image is shown in *f*.

10.2 Sample preparation: Cells

The cells used were obtained from the following sources:
NIH-3T3s (murine embryonic fibroblasts): Deutsche Sammlung von Mikroorganismen und Zellkulturen (DSMZ), DSMZ-no. ACC 59; **hMSC**s (human mesenchymal stem cells): Lonza, Switzerland, cat.-no. PT-2501; **C2C12** (murine myoblasts): DSMZ, DSMZ-no. ACC 565.
Cells were incubated at 37 °C at 5 % CO_2 in vent-cap culture flasks and on the day of splitting were washed at least once with Dulbecco's phosphate-buffered saline (DPBS) before trypsinization. Splitting was done every 4th day at most. Muscle-induced hMSCs (mi-hMSCs) were derived from naive hMSCs and remained attached up to 10 days. The medium was changed to muscle induction medium (MIM) as reported by Engler *et al.* (2, SM), more than a week before fixation.

10.3 Cryogenic protection of freeze-dried samples

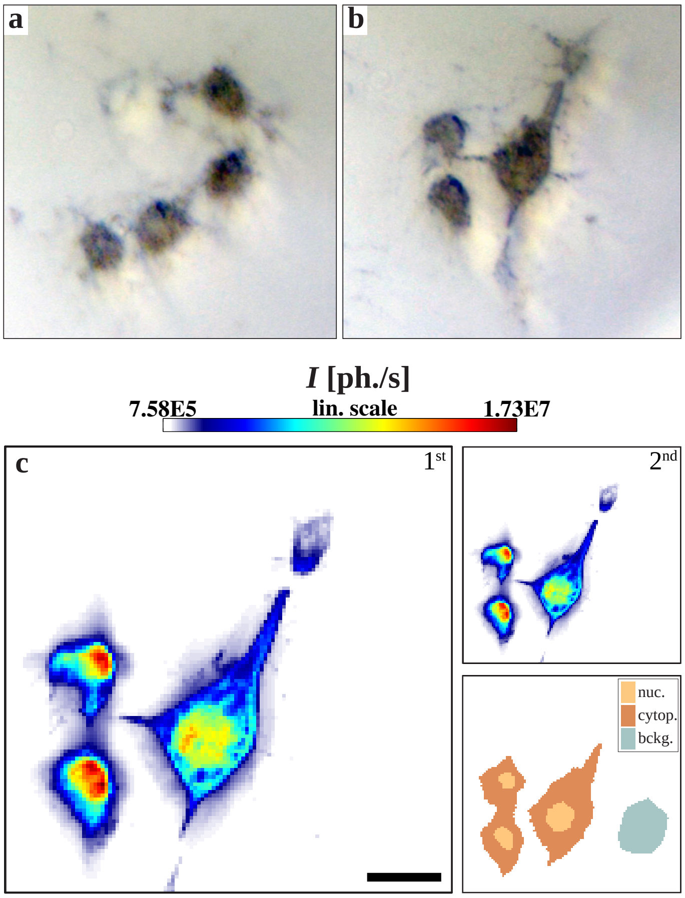

Figure 2: Optical micrographs of the scan areas (*a*) "5 cell"- and (*b*) "3 cell"-arrangement treated in Sec. 3 (main article). (*c*) First and second dark-field map of the "3 cell"-arrangement together with the stxm mask of the second scan series. Scalebar: 20 µm.

Towards establishing cryoprotection as a tool to preserve the diffraction signal of freeze-dried samples, two scan series were performed on adherent murine fibroblasts comprising three and two successive scans, respectively. Fig. 2 *a*-*b* show the OAV-images of the respective scan areas. *c* In agreement with the first scan series, x-ray dark-field images of the second scan series indicate no significant loss of scattering signal. In order to investigate the radial intensity profile $I(q_r)$, the corresponding first scan of both series was masked (see lower right corner) separating nucleic-, cytoplasmic- and background-regions. This mask was then transferred to all following scans adjusting translational alterations and differing stepsizes. After calculating an averaged 2D image for the nucleus-, cytoplasm- and background of each scan, data were transformed and substracted obtaining the background-corrected radial intensity profile for each scan, see Fig. 3 *a*-*b*. Data were then fitted by a power law function:

$$I(q_r) = a \cdot q_r^b + c \; . \tag{1}$$

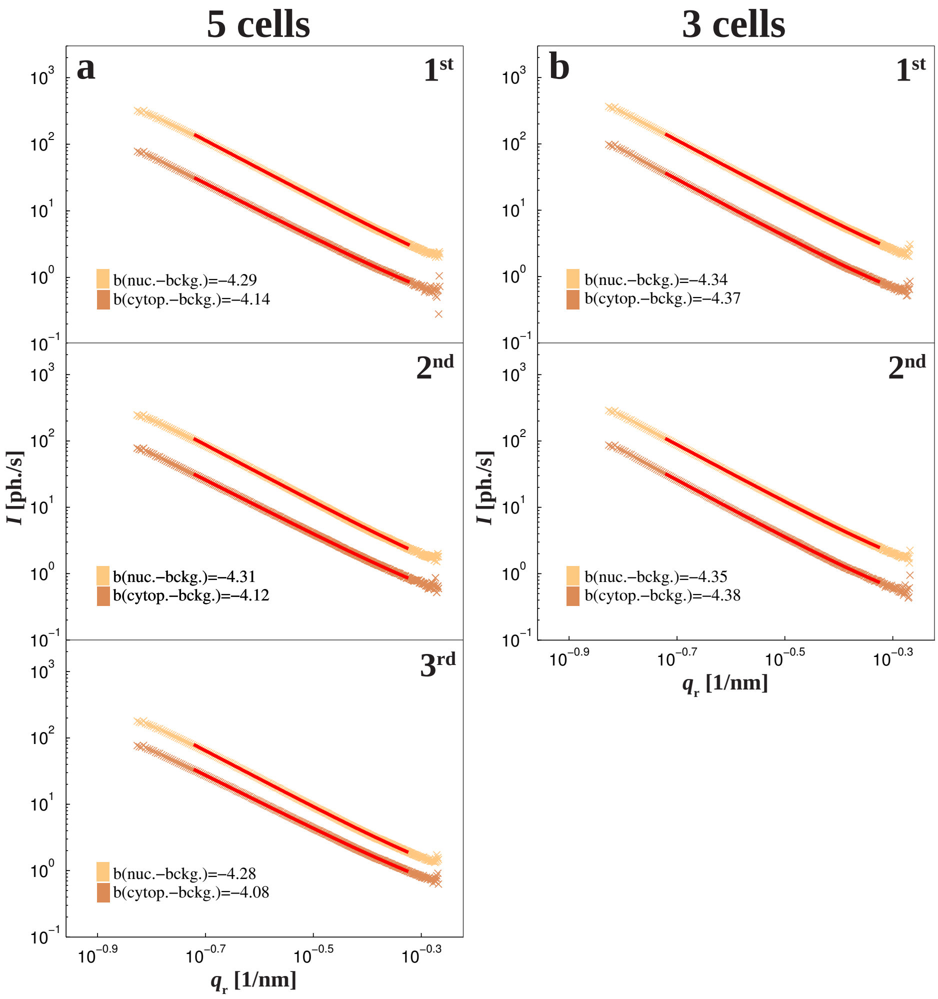

Figure 3: (*a*-*b*) After calculating a 2D-average signal, the respective background-corrected radial intensity profiles $I(q_\mathrm{r})$ were determined for each scan and fitted to a power law function fitted to the background-corrected signal of the nucleus (upper curve) and the cytoplasm (lower curve), respectively.

10.4 Sample Environment and Chambers

Wet sample experiments

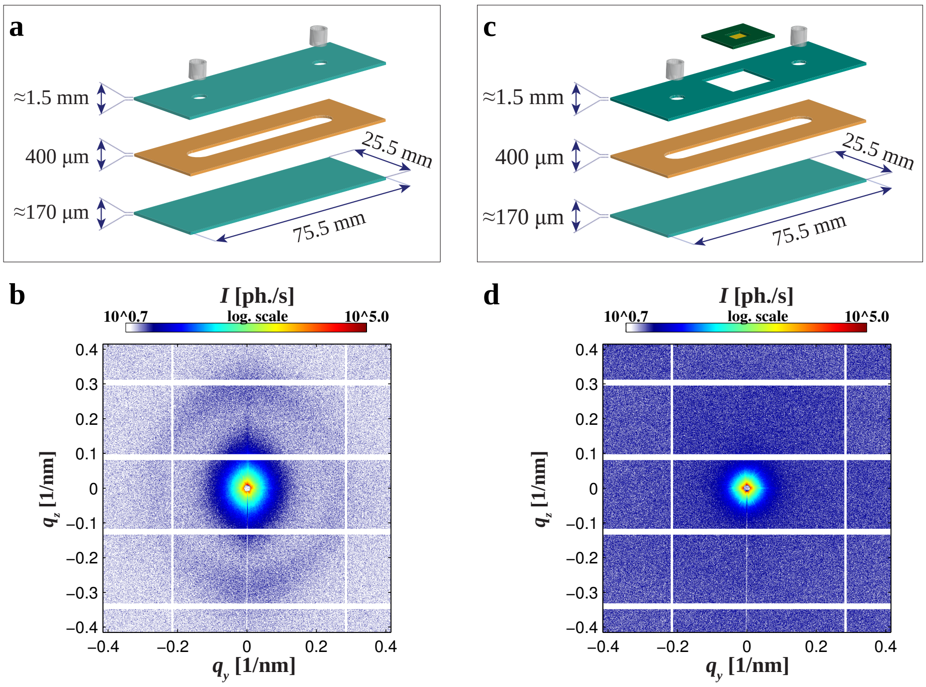

Figure 4: *left:* (*a*) Sketch of a commercial chamber (ibidi, Germany). (*b*) The diffraction signal of an equivalent 6-channel geometry sample (ibitreat, channel height: 400 µm, 6-channel cut) reveals a ring related to the polymer material of the upper slide. *right:* (*c-d*) When replacing the upper slide by highly transmissive Si_3N_4-windows this signal has vanished (ibitreat, channel height: 400 µm, 1-channel cut).

The development of sample environments that are simultaneously compatible with cell culture and x-ray microscopy is challenging. Previous approaches have used custom-built PMMA based microfluidic chambers in combination with ultrathin Si_3N_4-foils as x-ray windows onto which cells could adhere (8). Slides with microfluidic channels are commercially available for visible light microscopy (e. g., ibidi, Germany) and provide various biochemical coatings and physical surface treatments as well as sufficient gas exchange for the use of carbonate buffer (sketched in Fig. 4 *a*). We have tested these slides for the present x-ray nano-SAXS applications. Transmission at photon energy of $E_{\mathrm{ph.}} = 13.8\,\mathrm{keV}$ was considered satisfactory, and even at $E_{\mathrm{ph.}} = 8.7\,\mathrm{keV}$ acceptable, but the background of the SAXS patterns was found to be too high, despite the fact that the main polymer used is of low residual signal compared to other materials at similar thickness (5). In particular, our recordings reveal a characteristic peak at $q_r \approx 0.27\,\mathrm{nm}^{-1}$ (shown in *b*) caused by the upper slide of the polymer channel with an estimated thickness of about 1.5 mm. Together with the manufacturer we have developed an x-ray compatible design that includes an x-ray transparent and scattering free Si_3N_4-window (Silson, UK), replacing parts of the upper slide of the polymer channel (Fig. 4 *c-d*). Another chamber type with a sealed volume consists of two coverslips of borosilicate glass with an average thickness of ≈100 µm as window material and a punched piece of Parafilm serving as a spacer (not shown).

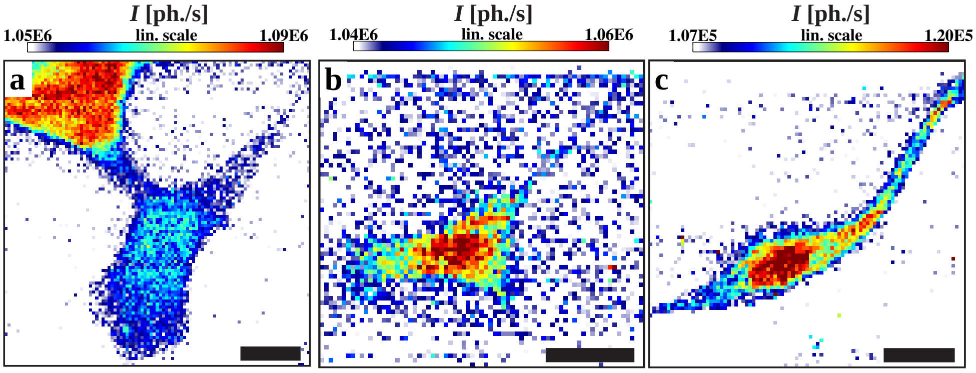

Figure 5: (*a*-*b*) X-ray dark-field images of murine myoblasts recorded in a commercially available chamber (ibidi, Germany). Scalebars: 10 µm and 20 µm. (*c*) Naive hMSC mounted on borosilicate glass and placed in an improvised glass-chamber. Scalebar: 20 µm.

Figure 5 shows nano-SAXS results obtained on hydrated fixed cells: In *a* & *b* the corresponding x-ray dark-field images are shown for murine myoblasts, cultivated in commercial cell culture slides (channel height: 200 µm, collagen IV coated bottom layer) and recorded at a photon energy of $E_{\mathrm{ph.}}$ =13.8 keV. Despite the elevated background and the reduced signal due to a smaller contrast between cells and solution, the cells can be clearly identified in the dark-field images. In *c*, the x-ray dark-field image is shown for a naive hMSC in a home built glass coverslip chamber, recorded at a photon energy of $E_{\mathrm{ph.}}$ =7.9 keV. The nucleus can be distinguished by eye, but the signal-to-noise ratio is too low to perform quantitative analysis on the diffraction data. For such low signals, even the slight intensity variations due to the top-up mode of the storage ring, i. e. the periodic refill of electrons, become noticable and must be corrected (not shown).

Frozen-hydrated samples

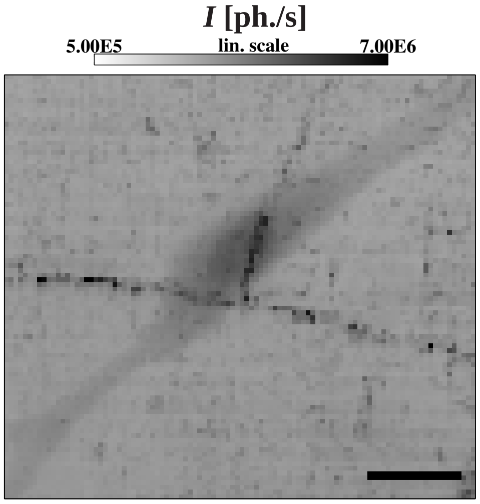

Figure 6: X-ray dark-field image of a frozen-hydrated naive hMSC. Scalebar: 20µm.

GINIX provides a cryogenic sample environment. Thus, one can avoid the invasive last step of freeze-drying eukariotic cells leaving them in a frozen-hydrated state.

Fig. 6 shows the x-ray dark-field image of a frozen-hydrated naive hMSC. The nucleus can be clearly distinguished from the cytoplasm. Two cracks show a strong overall scattering signal with pronounced anisotropy.

10.5 Structural differences among cell lines

Applying x-ray micro-diffraction on freeze-dried samples enables the quantification of local structures within a cell and a structural comparison among different cell lines. In this work, we propose ω obtained by PCA as a local and its respective mean Ω as a global order parameter describing anisotropy. Ω measures the overall anisotropic character, leading to relatively high values in case of a strong occurrence of parallel structures inside a cell. Data gathered so far indicate structural differences among cell lines. For a more detailed view, one representative scan for each cell line is chosen: See Fig. 7 for naive human mesenchymal stem cells, Fig. 6 (main article) for muscle-induced hMSCs, Fig. 8 for murine myoblasts and Fig. 9 for murine fibroblasts. All figures show the on-axis microscopic image (OAV) of the cell in *a* and the x-ray dark-field in *b*. After defining a region of interest, indicated by a black frame, a STXM mask derived from the dark-field image is applied on the corresponding PCA results, see *c*, as indicated by a pink contour line in *b*. The composite image is shown in *d* including all principal axes of the respective diffraction images.
In view of orientational variations within a cell, quantified by the nematic order parameter *s*, data reveal relatively low values for naive and muscle-induced hMSCs and relatively high values for murine myo- and fibroblasts. This finding becomes apparent when reviewing the PCA results of the representatives chosen. Fig. 10 shows the distribution of structure orientation angles θ_{pa} in a histogram. θ_{pa} covers a broad range of angles in case of mesenchymal stem cells while the distibution is narrow in the case of murine myo- and fibroblasts.

According to Liu *et al.* (6) *s* is derived by the second rank tensor *Q* as follows:

$$Q_{\alpha\beta} = \frac{1}{N_{\mathrm{ROI}}} \sum_{y,z|_{\mathrm{ROI}}} \left(2\, e_{\mathrm{pa},\alpha}(y,z)\; e_{\mathrm{pa},\beta}(y,z) - \delta_{\alpha\beta}\right)$$

$$Q = \frac{2}{N_{\mathrm{ROI}}} \cdot \begin{pmatrix} \sum\limits_{y,z|_{\mathrm{ROI}}} \left(e_{\mathrm{pa},1}(y,z) e_{\mathrm{pa},1}(y,z) - \frac{1}{2}\right) & \sum\limits_{y,z|_{\mathrm{ROI}}} e_{\mathrm{pa},1}(y,z) e_{\mathrm{pa},2}(y,z) \\ \sum\limits_{y,z|_{\mathrm{ROI}}} e_{\mathrm{pa},2}(y,z) e_{\mathrm{pa},1}(y,z) & \sum\limits_{y,z|_{\mathrm{ROI}}} \left(e_{\mathrm{pa},2}(y,z) e_{\mathrm{pa},2}(y,z) - \frac{1}{2}\right) \end{pmatrix}$$

$$\stackrel{*}{=} \frac{2}{N} \cdot \begin{pmatrix} \sum\limits_{y,z|_{\mathrm{ROI}}} \left((\cos(\theta_{\mathrm{pa}}(y,z)))^2 - \frac{1}{2}\right) & \sum\limits_{y,z|_{\mathrm{ROI}}} \cos(\theta_{\mathrm{pa}}(y,z)) \sin(\theta_{\mathrm{pa}}(y,z)) \\ \sum\limits_{y,z|_{\mathrm{ROI}}} \sin(\theta_{\mathrm{pa}}(y,z)) \cos(\theta_{\mathrm{pa}}(y,z)) & \sum\limits_{y,z|_{\mathrm{ROI}}} \left((\sin(\theta_{\mathrm{pa}}(y,z)))^2 - \frac{1}{2}\right) \end{pmatrix}$$

* assuming a general vector $\vec{e}_{\mathrm{pa}}(y,z) = \begin{pmatrix} e_1(y,z) \\ e_2(y,z) \end{pmatrix} = \begin{pmatrix} \cos(\theta_{\mathrm{pa}}(y,z)) \\ \sin(\theta_{\mathrm{pa}}(y,z)) \end{pmatrix}$ showing along the principal axis.

We then determined the eigenvalues $s_{1,2}$ of the equation:

$$Q \cdot \vec{b} = s \cdot \vec{b}$$

using Wolfram Mathematica 8 (Wolfram Research, IL, USA). The nematic order parameter is the positive eigenvalue $s_{1\vee 2} > 0$.

$$s_{1\vee 2} = \frac{1}{2N_{\mathrm{ROI}}} \left(-2N_{\mathrm{ROI}} + \sum_{y,z|_{\mathrm{ROI}}} 2(\cos(\theta_{\mathrm{pa}}(y,z)))^2 \right.$$

$$+ \sqrt{4\left(\sum_{y,z|_{\mathrm{ROI}}} 2\cos(\theta_{\mathrm{pa}}(y,z)) \sin(\theta_{\mathrm{pa}}(y,z))\right)^2 + \left(\sum_{y,z|_{\mathrm{ROI}}} 2\left(\cos(\theta_{\mathrm{pa}}(y,z))\right)^2 - \sum_{y,z|_{\mathrm{ROI}}} 2\left(\sin(\theta_{\mathrm{pa}}(y,z))\right)^2\right)^2}$$

$$\left. + \sum_{y,z|_{\mathrm{ROI}}} 2\left(\sin(\theta_{\mathrm{pa}}(y,z))\right)^2 \right)$$

$$\overset{(1)}{=} \frac{1}{2N_{\text{ROI}}} \left(\sqrt{4 \left(\sum_{y,z|_{\text{ROI}}} 2\cos(\theta_{\text{pa}}(y,z))\sin(\theta_{\text{pa}}(y,z)) \right)^2 + \left(\sum_{y,z|_{\text{ROI}}} 2\left(\cos(\theta_{\text{pa}}(y,z))\right)^2 - \sum_{y,z|_{\text{ROI}}} 2\left(\sin(\theta_{\text{pa}}(y,z))\right)^2 \right)^2} \right)$$

$$\overset{(2),(3)}{=} \frac{1}{2N_{\text{ROI}}} \sqrt{4 \left(\sum_{y,z|_{\text{ROI}}} \sin(2\theta_{\text{pa}}(y,z)) \right)^2 + \left(\sum_{y,z|_{\text{ROI}}} 4\cos(2\theta_{\text{pa}}(y,z)) \right)^2}$$

$$= \frac{1}{N_{\text{ROI}}} \sqrt{\left(\sum_{y,z|_{\text{ROI}}} \sin(2\theta_{\text{pa}}(y,z)) \right)^2 + \left(\sum_{y,z|_{\text{ROI}}} \cos(2\theta_{\text{pa}}(y,z)) \right)^2}$$

□

The results presented in section 6 (main article) are based on the datasets presented in Fig. 11 showing the OAV-image in the 1st and the x-ray dark-field image in the 2nd column. PCA results of the entire scan are separated showing the results of the order parameter ω in the 3rd and the orientation axis in the 4th column. The STXM mask used for statistical evaluation is depicted in the 5th column. Special attention was drawn on masking single cells which is important for determination of the nematic order parameter s. Parameters of each scan are listed in Tab. 1.

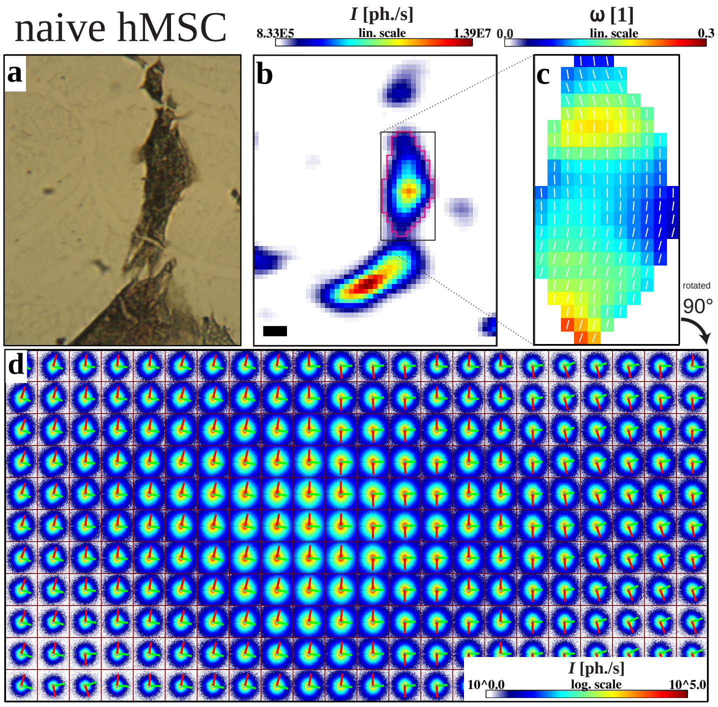

Figure 7: (*a*) OAV-image of freeze-dried naive hMSCs right before recording. (*b*) X-ray dark-field image using the micro-SAXS setup with a photon energy of $E_{\text{ph.}}$=8.7 keV). Scalebar: 40 µm. (*c*) PCA results of the marked region after applying a STXM mask, indicated as a pink contour line in *b*. (*d*) Composite image of the scan area shown in *c*. By PCA, two basis vectors are computed for every diffraction image.

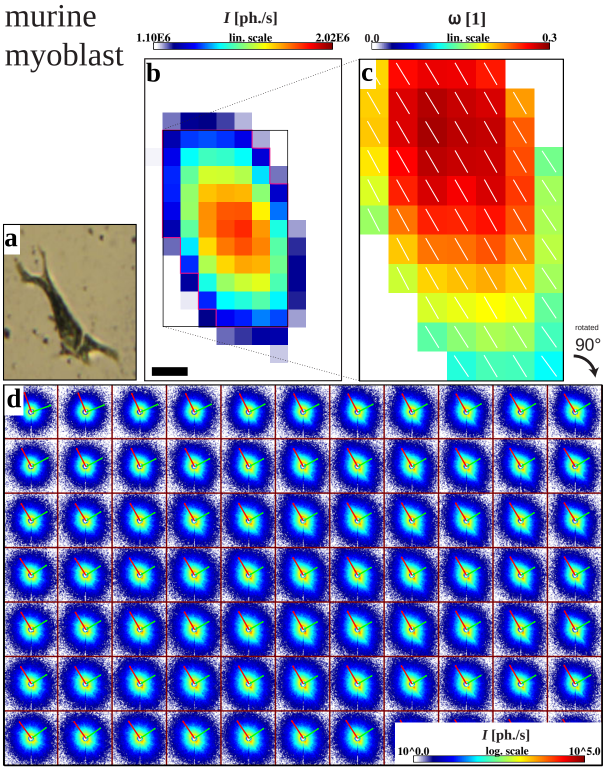

Figure 8: (*a*-*d*) equivalent arrangement as in Fig. 7, here on a murine myoblast. Scalebar: 10 µm.

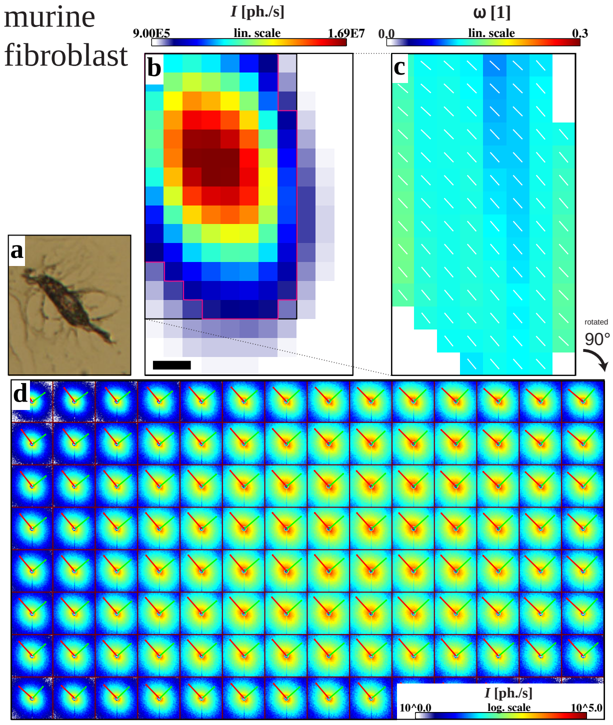

Figure 9: (*a*-*d*) equivalent arrangement as in Fig. 7, here on a murine fibroblast. Scalebar: 10 µm.

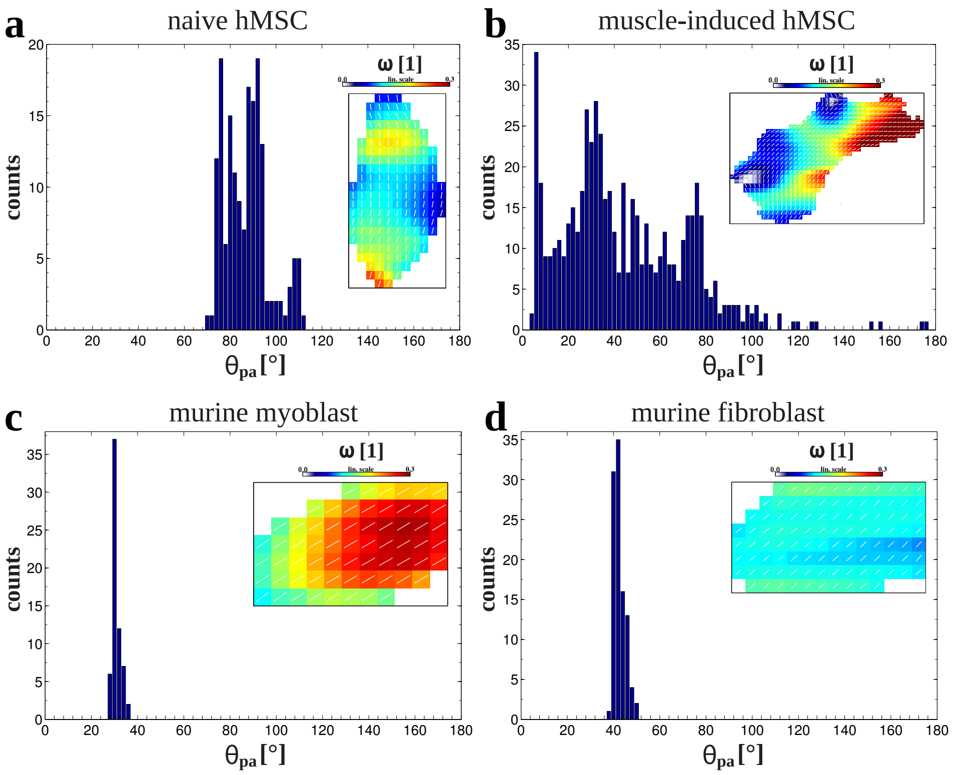

Figure 10: Histograms showing the distribution of structure orientation angles θ_{pa} in (*a*) a naive and (*b*) muscle-induced hMSC, (*c*) a murine myoblast and (*d*) a murine fibroblast.

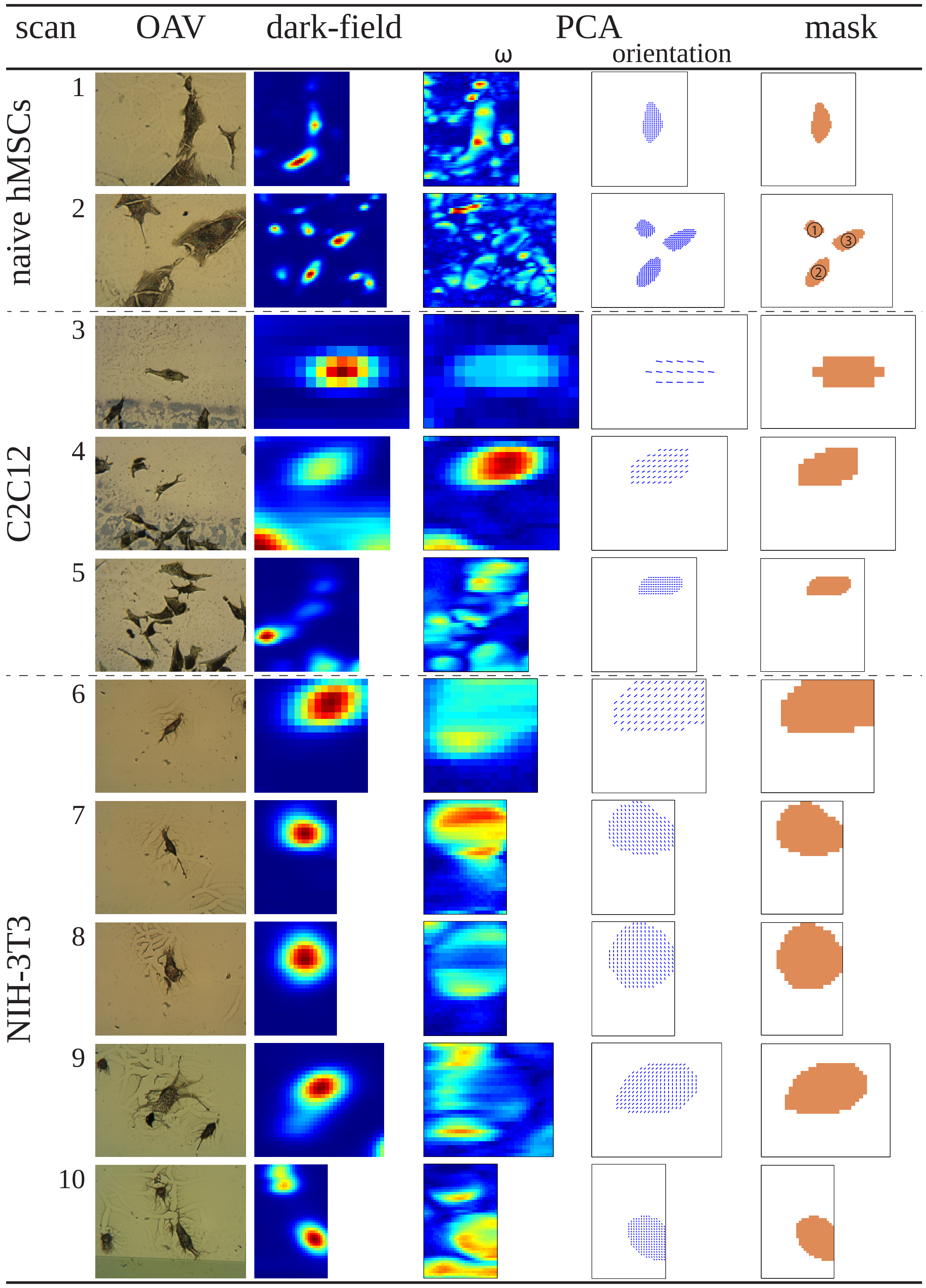
scan
OAV
dark-field
PCA
ω
orientation
mask
naive hMSCs
C2C12
NIH-3T3
1
2
3
4
5
6
7
8
9
10
①
②
③

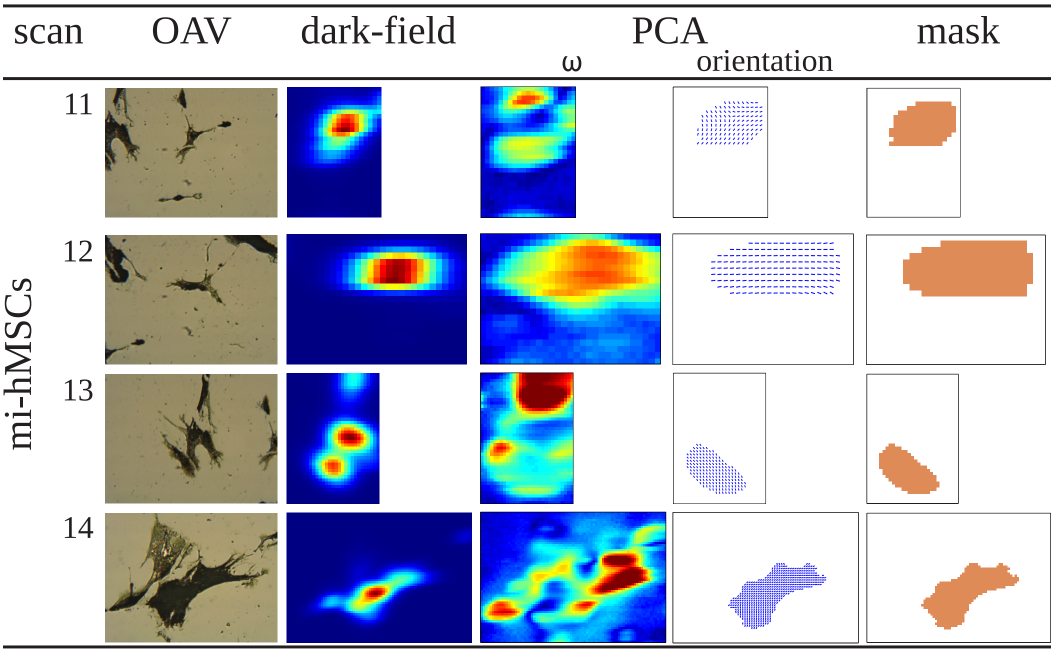

Figure 11: Overview of the datasets taken into account for statistical analysis showing the OAV-image in the 1st, the x-ray dark-field in the 2nd, the PCA order parameter and orientation in the 3rd and the 4th as well as the STXM masks for cells (red) in the 5th column. Note, that for presentation reasons individual limits were set for colorbars (not shown), so that a direct comparison between dark-fields is not applicable.

**cell type**	**naive hMSC**		**C2C12**			**NIH-3T3**					**mi-hMSC**			
**scan no**	**1**	**2**	**3**	**4**	**5**	**6**	**7**	**8**	**9**	**10**	**11**	**12**	**13**	**14**
Δ_y in µm	8	10	10	5	5	5	5	5	5	5	5	5	5	5
Δ_z in µm	8	10	10	5	5	5	5	5	5	5	5	5	5	5

Table 1: Scan step sizes for data shown in Fig. 7 (main article). All cells were grown on 1 µm-thick Si_3N_4-membranes, cryoprepared at alive state and then freeze-dried. All scans were performend at an energy of $E_{\text{ph.}} = 8.7\,\text{keV}$ with a dwell time of t_{dwell} =0.5 s and a flux of $I_0 = 1.44 \cdot 10^{11}$ ph./s using the instrumentation of the cSAXS endstation.

11 Bibliography

References

1. O. Bunk, M. Bech, T. H. Jensen, R. Feidenhans'l, T. Binderup, A. Menzel, and F. Pfeiffer. Multimodal x-ray scatter imaging. *New Journal of Physics*, 11(12): 123016, 2009.

2. A. J. Engler, S. Sen, H. L. Sweeney, and D. E. Discher. Matrix elasticity directs stem cell lineage specification. *Cell*, 126(4):677–689, 2006.

3. B. Henrich, A. Bergamaschi, C. Broennimann, R. Dinapoli, E.F. Eikenberry, I. Johnson, M. Kobas, P. Kraft, A. Mozzanica, and B. Schmitt. Pilatus: A single

photon counting pixel detector for x-ray applications. *Nuclear Instruments and Methods in Physics Research Section A: Accelerators, Spectrometers, Detectors and Associated Equipment*, 607(1):247 – 249, 2009. Radiation Imaging Detectors 2008Proceedings of the 10th International Workshop on Radiation Imaging Detectors.

4. S. Kalbfleisch, H. Neubauer, S. P. Krüger, M. Bartels, M. Osterhoff, D. D. Mai, K. Giewekemeyer, B. Hartmann, M. Sprung, and T. Salditt. The Göttingen Holography Endstation of Beamline P10 at PETRA III/DESY. *AIPConf.Proc.*, 1365(1):96–99, 2011.

5. Lawrence Berkeley National Laboratory. The center for x-ray optics (cxro). last accessed on 04/01/15. URL `http://www.cxro.lbl.gov/`.

6. B. Liu, T. H. Besseling, M. Hermes, A. F. Demirors, A. Imhof, and A. van Blaaderen. Switching plastic crystals of colloidal rods with electric fields. *Nat Commun*, 5:3092, 2014.

7. T. Salditt, S. Kalbfleisch, M. Osterhoff, S. P. Krüger, M. Bartels, K. Giewekemeyer, H. Neubauer, and M. Sprung. Partially coherent nano-focused x-ray radiation characterized by Talbot interferometry. *Opt. Express*, 19(10):9656–9675, May 2011.

8. B. Weinhausen and S. Köster. Microfluidic devices for x-ray studies on hydrated cells. *Lab Chip*, 13:212–215, 2013.
