# Supplementary material for: X-Ray Micro- and Nanodiffraction Imaging on Human Mesenchymal Stem Cells and Differentiated Cells
Source: Biophys J. 2016 Feb 2;110(3):680–90. doi: 10.1016/j.bpj.2015.12.017 (PMC4744168; doi:10.1016/j.bpj.2015.12.017)
Supplement: Document S2. Article plus Supporting Material [file mmc2.pdf]

Biophysical Journal Volume 110 February 2016 680–690

Article

X-Ray Micro- and Nanodiffraction Imaging on Human Mesenchymal Stem Cells and Differentiated Cells

Marten Bernhardt,[1] Marius Priebe,[1] Markus Osterhoff,[1] Carina Wollnik,[2] Ana Diaz,[3] Tim Salditt,[1,*] and Florian Rehfeldt[2,*]

[1]Institut für Röntgenphysik, Georg-August-Universität Göttingen, Göttingen, Germany; [2]Drittes Physikalisches Institut - Biophysik, Georg-August-Universität Göttingen, Göttingen, Germany; and [3]Paul Scherrer Institut, Villigen, Switzerland

ABSTRACT Adult human mesenchymal stem cells show structural rearrangements of their cytoskeletal network during mechanically induced differentiation toward various cell types. In particular, the alignment of acto-myosin fibers is cell fate-dependent and can serve as an early morphological marker of differentiation. Quantification of such nanostructures on a mesoscopic scale requires high-resolution imaging techniques. Here, we use small- angle x-ray scattering with a spot size in the micro- and submicrometer range as a high-resolution and label-free imaging technique to reveal structural details of stem cells and differentiated cell types. We include principal component analysis into an automated empirical analysis scheme that allows the local characterization of oriented structures. Results on freeze-dried samples lead to quantitative structural information for all cell lines tested: differentiated cells reveal pronounced structural orientation and a relatively intense overall diffraction signal, whereas naive human mesenchymal stem cells lack these features. Our data support the hypothesis of stem cells establishing ordered structures along their differentiation process.

INTRODUCTION

Human mesenchymal stem cells (hMSCs) provide a source for a broad spectrum of cell types for regeneration (1,2). Induced by biochemical signals (3) as well as by the mechanical properties of the surrounding tissue (4–7), multipotent hMSCs undergo differentiation toward terminally differentiated and thus specialized cells. Besides changes in gene regulation, hMSCs also reorganize their acto-myosin network during this process. These structural changes are cell fate-dependent, ranging from randomly oriented to parallel fiber bundles (4). An intimate understanding of the structural change as a function of cell fate is essential to improve stem cell treatment—possibly without the need of external biochemical induction—and might enable new approaches in medicine (8,9).

Visible light microscopy with its specific labeling capability is arguably the most important tool to visualize a specific protein network, fundamentally enhanced by the recent superresolution capabilities, such as stimulated emission depletion microscopy (10,11) or stochastic switching microscopy (12–14). However, the strength of fluorescence microscopy—specific labeling on a molecular level—is also a weakness, as unlabeled cellular constituents remain invisible. Therefore, complementary imaging methods capable to probe the native unlabeled density distribution are needed. To this end, small-angle x-ray scattering (SAXS), which is commonly applied to study homogeneous macromolecular structures in macroscopic solutions or suspensions (15–18), has been recently introduced in cellular imaging, by focusing the beam down to the subcellular scales (19–22). In this manner, every diffraction pattern of a given scan over the cells contains the corresponding local structural information. Depending on the range of scattering vectors, also known as momentum transfer, structures down to molecular scales become accessible (22). Recent proof-of-concept experiments and applications included scanning nanodiffraction of keratin-enriched human carcinoma cells, studied in a correlative manner by visible light fluorescence (20), and bacteria (19,23). The latter were enhanced by coherent x-ray imaging providing an inversion of the local diffraction image and hence a superresolution image of the electron density distribution with a resolution better than the beam size. This method can even be applied to living cells with some restrictions, concerning in particular radiation damage and signal/noise (21,22). A nano-SAXS study of *Dictyostelium discoideum* revealed pronounced anisotropic scattering on the perimeter of the cell with a predominant orientation of diffraction streaks perpendicular to the plasma membrane, attributed to actin fiber bundles oriented parallel to the membrane, also known as the actomyosin cortex (22). Similar streaklike diffraction patterns were observed in frozen-hydrated suspensions, i.e., thin vitrified films, of in vitro F-actin, cross linked with α-actinin, indicating highly ordered bundles of filaments.

In this work, we use micro- and nanofocus SAXS, in the following denoted as micro- and nano-SAXS, to study naive

Submitted August 12, 2015, and accepted for publication December 7, 2015.

*Correspondence: tsaldit@gwdg.de or florian.rehfeldt@phys.uni-goettingen.de

This is an open access article under the CC BY license (http://creativecommons.org/licenses/by/4.0/).

Editor: Christopher Yip.

© 2016 The Authors

0006-3495/16/02/0680/11

http://dx.doi.org/10.1016/j.bpj.2015.12.017

CrossMark

hMSCs, biochemically induced hMSCs driven toward the myogenic lineage, in the following denoted as muscle-induced hMSCs (mi-hMSCs), murine myoblasts (C2C12), and murine embryonic fibroblasts (NIH-3T3) in a freeze-dried state. Freeze-drying reduces the specimen to the nonvolatile components and yields a high signal/noise. Therefore, this state is well suited to develop the technique before translation to the physiologically more relevant aqueous state. Results presented here have been obtained using two different synchrotron beamlines: nano-SAXS was performed at the coherent nanofocus endstation Göttingen Instrument for Nano-Imaging with X-Rays at the P10 beamline at the PETRA III storage ring at DESY in Hamburg, Germany, and micro-SAXS was performed at the cSAXS beamline at the Swiss Light Source at the Paul Scherrer Institut in Villigen, Switzerland. Our main hypothesis is that the electron density variations in naive stem cells are comparatively low, indicative of a weak structural organization, while the differentiated cells may exhibit stronger Fourier components, as expected for cell types that exhibit higher structural order of their cytoskeleton, e.g., myoblasts.

Fig. 1 *a* illustrates the basic concept of SAXS recordings with focused beams: an undulator-generated x-ray beam is monochromatized and focused. While the primary beam is blocked, scattered photons are recorded in the far field using a two-dimensional (2D) single photon counting detector. After defining a suitable region of interest via an on-axis video (OAV) microscope, the sample is scanned using a piezo stage. The result is an array of diffraction images, which can be converted to a real space map of different structural observables. In the simplest case, the scattered photons are integrated for each 2D diffraction image, resulting in an x-ray dark field image of the sample $I_{\text{diffraction}}(y,z)$ as a function of the relative scan positions y and z. Representative dark field maps are shown in Fig. 1 *b* (freeze-dried murine fibroblasts recorded with the nano-SAXS setup) and in Fig. 1 *c* (freeze-dried naive hMSCs recorded with the micro-SAXS setup). Samples are aligned and monitored during the x-ray scans by the OAV; see Fig. 1 b (*upper right*). When comparing the two datasets, the higher real space resolution of the nano-SAXS scan (0.5 μm step size) compared to the micro-SAXS dataset (8 μm step size) becomes immediately apparent. Although the beamsize limits the real space resolution on the one hand, one must keep in mind that relaxed focusing conditions, resulting in an almost parallel propagating beam, may enable cleaner and more highly resolved diffraction patterns, as well as lower local dose on the other hand.

This article is organized as follows: after this Introduction and the setup conditions described in Materials and Methods, a method to reduce radiation damage in cells by

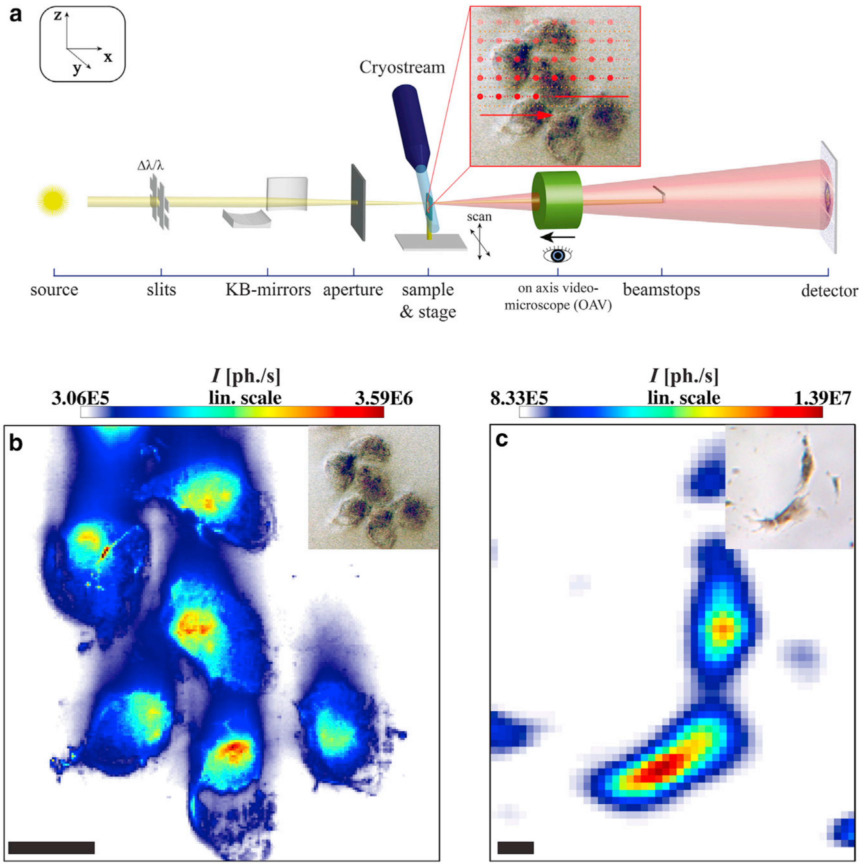

FIGURE 1 (*a*) Schematic of major optical components of a SAXS experiment with a nanofocused beam. (*b*) X-ray dark field image of lyophilized murine fibroblasts on glass, recorded with the nano-SAXS setup. Micrograph of the sample from the on-axis microscope, right before scanning (*inset*). Scale bar, 20 μm. (*c*) X-ray dark field image of lyophilized naive hMSCs on Si_3N_4-membranes recorded with a micro-SAXS setup and micrograph of the sample before scanning (*inset*). Scale bar, 40 μm. To see this figure in color, go online.

a cryogenic sample protection is presented in Cryogenic Protection of Samples. Nano- and micro-SAXS results on freeze-dried samples are shown in Freeze-Dried Samples and Structure Factor with an emphasis on anisotropies in the diffraction patterns. Analysis of Anisotropy in the Diffraction Patterns then describes an automated empirical analysis of the diffraction patterns based on principal component analysis (PCA). A comparison of the structural parameters derived for all cell lines is given in Comparison of Different Cell Types, pointing at pronounced cell type-specific differences, before the article closes with a brief summary and Conclusion.

MATERIALS AND METHODS

Sample preparation

Substrates

hMSC, mi-hMSC, NIH-3T3, and C2C12 were prepared on Si_3N_4-ultrathin membranes (Silson, Warwickshire, UK) of 1 μm-thickness or glass coverslips of $\approx$100 μm-thickness. Membranes and glass coverslips were plasma-cleaned before preparation. A drop of medium was pipetted on top of the substrate at room temperature, followed by a second drop after more than 5 min. The substrate was rinsed with 2 mL of Dulbecco's phosphate-buffered saline (DPBS). After detachment and washing of the cell-stock, a droplet of the cell suspension of typically 20 μL (100–200 cells/μL) was brought onto the partially wet and coated substrate. Cells settled for ~10 min at room temperature. This step was carried out two to three times. After settlement, the samples were immersed in 2 mL of prewarmed nutrition medium, and incubated at 37°C/5%CO_2 for up to 5 days. For C2C12-cells in a commercially available chamber cell, suspension (40 cells/μL) was brought into a collagen IV-coated μ-slide with a channel height of 200 μm. Sample was fixed the next day. The self-assembled glass chamber was formed using a second coverslip. A hole-punched parafilm segment was placed on top. A droplet of DPBS was placed into the hole in the center. Next, the cell-containing glass coverslip was placed upside-down, and the parafilm was locally heated forming a tight chamber. The chamber was finally sealed using nail polish.

Cells

See Section S10.2 in the Supporting Material.

Sample fixation

Cryofixation. The samples were imaged by phase contrast microscopy right before plunging. The substrates were blotted manually, plunged using the commercially available grid plunger model EM GP (Leica Microsystems, Wetzlar, Germany), and stored in liquid nitrogen thereafter. Lyophilization was done in a home-built freeze-drier for 3 days and all samples were transferred to silica gel-filled desiccators where they remain until shortly before the x-ray measurements.

Chemical fixation. Samples were fixed using prewarmed 9% formaldehyde in DPBS. All samples were washed three times and stored in DPBS in the refrigerator.

Instrumentation

Nanofocus setup

Nano-SAXS data were recorded at the P10-beamline of DESY's PETRAIII storage ring, using the Göttingen Instrument for Nano-Imaging with X-Rays (24). Freeze-dried and chemically fixed samples in glass chambers were recorded at a photon energy of E_{photon} = 7.9 keV (P10/first run). The beam was focused by a pair of Kirkpatrick-Baez (KB) mirrors to a size of $\approx$250 $\times$ 320 nm (vertical $\times$ horizontal, full width at half-maximum (FWHM)) as measured by translation of a waveguide. Two soft-edge apertures were placed in front of the sample to block parasitic x-rays from the mirrors (25). The focused photon flux measured at the detector was $I_0 = 1.29 \times 10^{11}$ photon/s. The beam could be attenuated using a set of aluminum foils. The sample was mounted on a motorized stage and placed in the focus at $\approx$20 cm downstream from the KB-mirrors. An OAV microscope with 10–30 $\times$ optical zoom, a working distance of 50 mm, and LED illumination was used for alignment and to define suitable scan regions. Optionally, cryogenic conditions could be applied to the sample via a cryogenic nitrogen gas stream (Oxford Instruments, Abingdon, Oxfordshire, UK). The diffraction patterns for each scan point were recorded by the single photon counting pixel detector Pilatus 300k (Dectris, Baden, Switzerland), positioned $\approx$5.1 m behind the focal plane. To reduce absorption, the beam was guided through an evacuated tube. The primary beam was blocked by multiple semi-transparent beamstops. Scans were performed in a discrete or continuous mode with a step size of typically Δ_{real} = 0.5–1 μm, defining the real space resolution. In the discrete scanning mode, the beam was blocked by a fast shutter when moving the sample, while in continuous mode the beam shutter remained open when scanning along the fast axis, i.e., along the y direction. Recordings of hydrated cells in commercial cell culture chambers were performed at E_{photon} = 13.8 keV (P10/second run) with a maximum flux of $I_0 = 1.38 \times 10^{11}$ photons/s measured at the detector and a focus size of $\approx$180 $\times$ 370 nm (vertical $\times$ horizontal, FWHM) as measured by translation of a waveguide. A set of molybdenum foils was used for attenuation, all other parameters were comparable to the P10/first run.

Microfocus setup

Micro-SAXS data were recorded at the cSAXS-beamline of the Swiss Light Source. The x-ray beam was focused by a Si(111) crystal monochromator in the horizontal and a Rh-coated mirror in the vertical direction resulting in a photon energy of E_{photon} = 8.7 keV and a beamsize of ~33 $\times$ 54 μm (vertical $\times$ horizontal, FWHM) at the sample position. The primary flux measured at the detector was $I_0 = 1.44 \times 10^{11}$ photon/s. The sample was positioned in the focal plane at $\approx$5 m downstream from the mirrors. Multiple step motors and a hexapod enabled the translation of the sample; the step size varied from typically 5–10 μm. A 10$\times$ on-axis optical microscope was used for alignment and surveillance of the sample. The shape and size of the beam at the sample position were determined by a scintillator based x-ray-microscope. An evacuated tube of 7 m length spans the distance from the sample to the single photon counting pixel detector Pilatus 2M (26).

For an overview of the fundamental setup parameters, see Table 1.

RESULTS

Cryogenic protection of samples

Cells within the scan area of the focused beam suffer from high radiation dose, causing structural damage. The radiation dose applied in every scan point is given by

TABLE 1 Setup Parameters of the X-Ray Experiments

Beamline	Beam Diameter		E (keV)	λ (pm)	I_0 (photons/s)
	Horizontal	Vertical			
DESY/PETRAIII/P10 (first run)	320 nm	250 nm	7.9	156.9	1.29×10^{11}
DESY/PETRAIII/P10 (second run)	370 nm	180 nm	13.8	89.8	1.38×10^{11}
PSI/SLS/cSAXS	54 μm	33 μm	8.7	142.5	1.44×10^{11}

$$D = \frac{I_0 \times \tau \times E_{\text{photon}}}{l_{\text{attenuation}} \times \rho \times \Delta_y \times \Delta_z}, \quad (1)$$

with the intensity I_0 in photons/s, dwell time τ in s, photon energy $E_{\text{photon}} = 7.9$ keV, the focal spot size Δ_y and Δ_z (FWHM), and the attenuation length $l_{\text{attenuation}} = 7.2 \times 10^{-4}$ m evaluated for a hypothetical model protein $H_{50}\ C_{30}\ N_9\ O_{10}\ S$ with a given density $\rho = 1.35$ g/cm^3 (27). Radiation damage at high dose can, for example, manifest itself by changes in the radial intensity profile $I(q_r)$ and an overall reduction in the scattering intensity in subsequent scans on the same area. One well-known approach to minimize radiation damage is to use cryogenic sample conditions. According to Meisburger et al. (28), cryo-cooled proteins and nucleic acids can withstand doses at least two orders-of-magnitude larger than samples at room temperature. To verify that radiation damage did not affect the recorded signals in a significant way under cryogenic conditions, we performed a scan series on freeze-dried murine fibroblasts applying a dose of $D \approx 2.1 \times 10^8$ Gy in each of three successive scans, while keeping the sample in a cryogenic gas stream (Oxford Instruments). As shown in Fig. 2, *a–c*, the contrast images are indeed perfectly consistent and without any significant structural alterations. For the x-ray dark field value shown in each of the image pixels, the diffraction signal was integrated over the detector, excluding primary beam {pb} and setup-related parasitic scattering {ps}

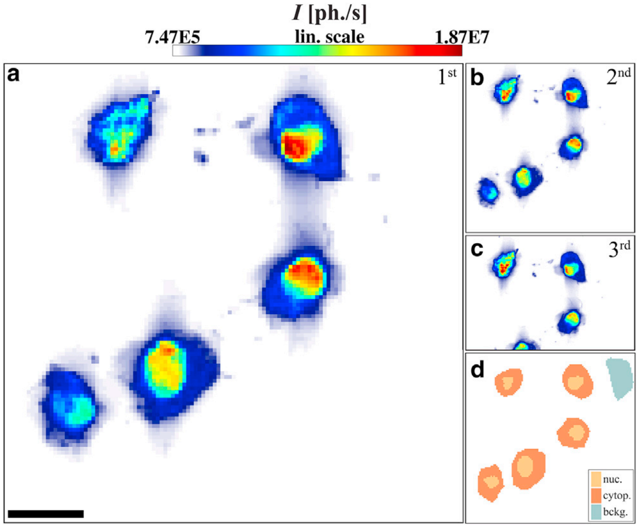

FIGURE 2 Images of five freeze-dried murine fibroblasts. (*a–c*) X-ray dark field maps of lyophilized murine fibroblasts on a Si_3N_4-membrane recorded with a nanofocused beam with samples kept in a cryogenic gas stream to suppress radiation damage. Three subsequent scans (*a*, *b*, and *c*) are recorded, the first two with a step size of $\Delta_{y,z} = 1$ μm, the last with $\Delta_{y,z} = 0.5$ μm. Scale bar, 20 μm. (*d*) Dark field mask, defining nucleic and cytoplasmic regions for further quantification. To see this figure in color, go online.

$$I_{\text{diffraction}}(y,z) = \int_{\mathbb{R}^2 \setminus [\{pb\} \cup \{ps\}]} I(y,z,q_y,q_z)\,dq_y\,dq_z, \quad (2)$$

with q_y and q_z components of the scattering vector in the *y-z* plane.

To further quantify possible structural alterations, we have evaluated the radial intensity profiles of the scans and averaged over all pixels in a certain cellular region, as defined by a software mask separating cytoplasms and nuclei (Fig. 2 d). After calculating the corresponding averaged 2D diffraction image, an azimuthal average is determined to yield a one-dimensional radial intensity profile $I(q_r)$. Profiles are fitted to a power-law decay, after subtraction of a constant background signal (22)

$$I(q_r) = a \times q_r^b + c\,, \quad (3)$$

with the scattering vector component $q_r = \sqrt{q_y^2 + q_z^2}$ perpendicular to the primary beam on the detector plane. Results of the scan series are listed in Table 2: one five-cell arrangement recorded by three subsequent scans and one three-cell arrangement recorded by two subsequent scans (see Section S10.3 in the Supporting Material). They reveal only slight deviations in the exponent *b* (see Table 2), which are not significant in view of the statistical and systematic errors of the procedure. In particular, we do not observe a steepening in the power law decay, as observed in Priebe et al. (22) for samples suffering from beam-induced damage. We conclude that cryoprotection preserves freeze-dried cells sufficiently for nano-SAXS scans.

Freeze-dried samples and structure factor

We have performed multiple scans on freeze-dried samples using the nano- and micro-SAXS setup. We first show nano- and then micro-SAXS results, illustrating typical data that can be obtained in scanning SAXS experiments on cells, and simple approaches to quantify the radial intensity decay. Even if not yet analyzed with a model, the quality of the diffraction data underlines the potential of the label-free imaging technique to reach high resolution even in weakly scattering cells. Fig. 3 shows a representative nano-SAXS dataset of a freeze-dried mi-hMSC mounted on a Si_3N_4-membrane, recorded with a step size of $\Delta_{y,z} = 0.5$ μm. A phase contrast visible light micrograph of the cell (40×) before cryo-plunging is shown in Fig. 3 *a*. The region indicated by the red rectangle corresponds to the approximated scanning region with its nano-SAXS dark field map shown in Fig. 3 *b*, revealing position and shape of the cell with its nucleus, which can be distinguished from the cell's periphery, i.e., the cytoplasm, by a change in scattering cross section. To identify anisotropies in the diffraction patterns, the so-called streakfinder algorithm (22) is applied, and the orientation is plotted for all pixels with anisotropy exceeding

TABLE 2 Fit Results for Cryoprotected Scan Series

Scan	Region of Interest	Run	*a* ((photons × nm)/s)	*b* (1)	*c* (photons/s)	R^2 (1)
Five cells	cytop-bckg	first	3.2×10^{-2}	−4.14	1.5×10^{-1}	0.9998
		second	3.3×10^{-2}	−4.12	1.3×10^{-1}	0.9998
		third	3.8×10^{-2}	−4.08	1.7×10^{-1}	0.9998
	nuc-bckg	first	1.1×10^{-1}	−4.29	3.0×10^{-1}	0.9996
		second	8.4×10^{-2}	−4.31	2.9×10^{-1}	0.9996
		third	6.5×10^{-2}	−4.28	3.2×10^{-1}	0.9997
Three cells	cytop-bckg	first	2.5×10^{-2}	−4.37	1.7×10^{-1}	0.9997
		second	2.2×10^{-2}	−4.38	1.6×10^{-1}	0.9997
	nuc-bckg	first	1.0×10^{-1}	−4.34	4.9×10^{-1}	0.9999
		second	7.9×10^{-2}	−4.35	4.4×10^{-1}	0.9999

cytop-bckg, cytoplasmic signal minus background signal; nuc-bckg, nucleic signal minus background signal.

a selected threshold (parameters $S > 0.09$ as defined in Priebe et al. (22)). Regions of consistent orientation can be recognized: neighboring pixels reveal a similar orientation while the overall orientation coincides with the expectation that the stress fiber alignment is predominantly along the extensions of adherent cells (4). Pronounced anisotropies occur close to the plasma membrane; see single diffraction image in Fig. 3 *d*, located at the position marked in Fig. 3 *c*.

Nanodiffraction allows us to investigate highly localized structures in the cell, probed in each diffraction point. However, focusing is accompanied by diffraction broadening of the primary beam behind the sample, decreasing the angular resolution Δq in the detection plane. We therefore complement the nano-SAXS data by micro-SAXS recordings with lower resolution in real space, but higher resolution in reciprocal space, and moreover less parasitic scattering, because the focusing is relaxed. This is implemented at the cSAXS beamline, providing an isotropic and clean direct beam signal in the far field without the typical artifacts of KB focusing. Fig. 4 shows one example of a scan performed on freeze-dried murine myoblasts on a Si_3N_4-window. The x-ray dark field image reveals high scattering intensity in the center of the cell (Fig. 4 *a*). The beam size, which is in the same order of the cell size, blurs the real-space image and the contour of the nucleus and the cell membrane are not clearly seen. The clean diffraction signal enables PCA (see Fig. 4 *b*). Local anisotropies can be quantified by definition of an anisotropy parameter ω (color-coded), as detailed in Analysis of Anisotropy in the Diffraction Patterns. Orientations of the spatial structures are perpendicular to the scattering signal and are

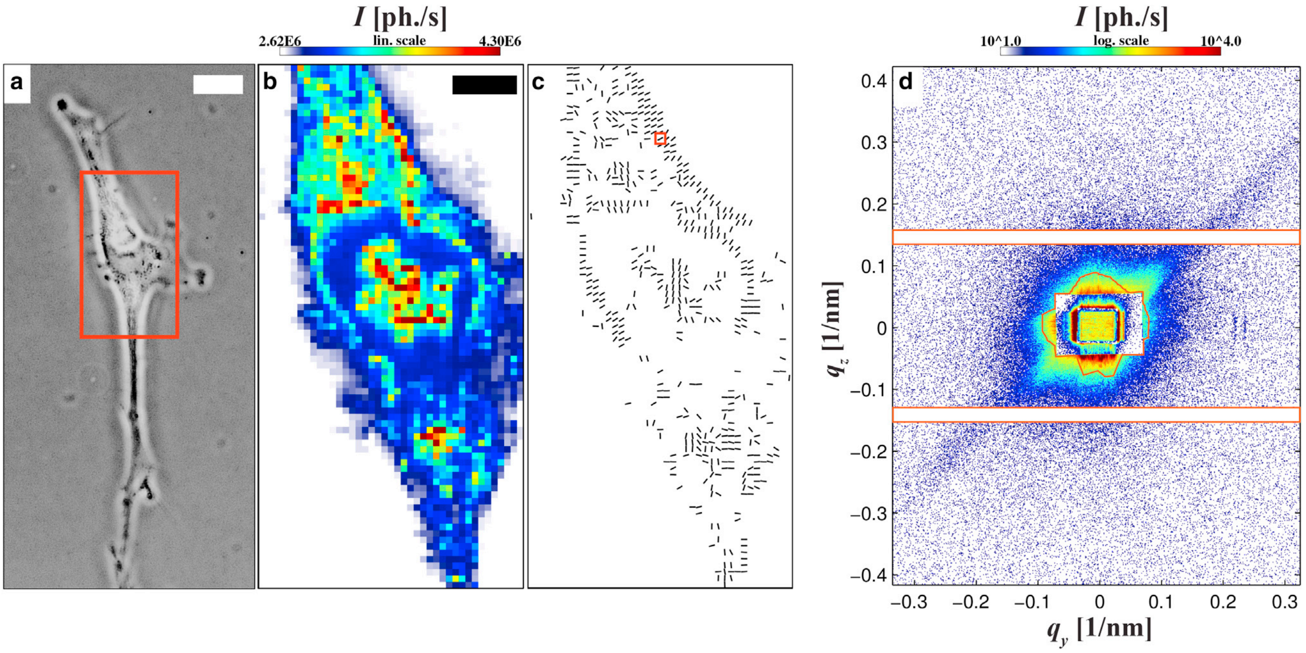

FIGURE 3 (*a*) A 40× phase contrast image of a mi-hMSC recorded right before cryo plunging. The red frame shows the approximated scan region. Scale bar, 20 μm. (*b*) X-ray dark field map recorded with the nano-SAXS setup. Scale bar, 5 μm. (*c*) Anisotropic diffraction, evaluated as in Priebe et al. (22), visualizing the anisotropy in terms of the direction of the diffraction peak. (*d*) Single diffraction image at the position marked in (*c*) also showing rectangular shadows of the semitransparent beamstops in the center. The detector mask is indicated as orange frames. To see this figure in color, go online.

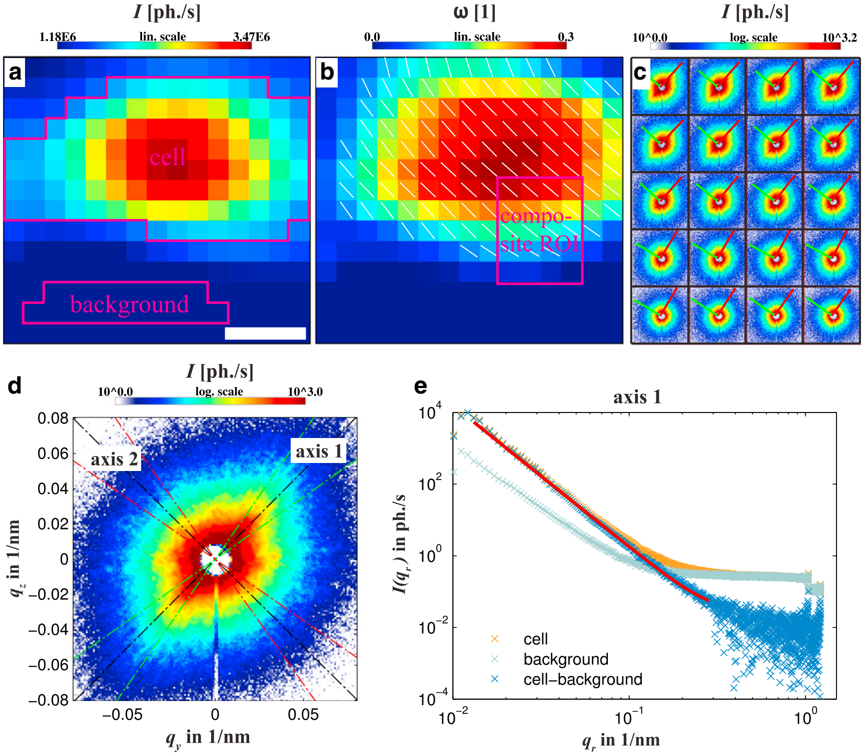

FIGURE 4 (*a*) X-ray dark field image of a freeze-dried murine myoblast recorded with the micro-SAXS setup. Scale bar, 20 μm. (*b*) PCA result: White lines indicate the principal orientation axis. The anisotropy parameter ω (see Eq. 9) is color-coded. (*c*) Composite of the region marked in (*b*). Each diffraction pattern is cropped to a region of $q_r < 0.11$ nm^{-1}. The corresponding eigenvectors are scaled by their relative standard deviation and depicted as red and green arrows (for further explanations, see Analysis of Anisotropy in the Diffraction Patterns). (*d*) Average diffraction signal of the cell. The region taken into account is marked in (*a*). The two axes resulting from PCA indicate the principal directions of anisotropy, the directions of lowest and highest variance. (*e*) Averaged radial intensity profile of a segment $\pm 10°$ around axis 1. Data are then fitted by a power law function following Eq. 4, resulting in $b = -3.95$ (axis 1) and $b = -3.74$ (axis 2), not shown. To see this figure in color, go online.

depicted as white lines. In agreement with the composite image (Fig. 4 *c*), pronounced and consistent anisotropy is found throughout the entire cell, with a maximum in its center. To obtain additional structural information, an average cell diffraction signal is computed, onto which PCA is applied (Fig. 4 *d*). The PCA yields the two principal (orthogonal) axes $k \in [1, 2]$ with corresponding standard deviation of the photons' momentum transfer σ_k as a model-free parameter for the scattering distribution, here $\sigma_1 = 1.9 \times 10^{-2}$ nm^{-1} and $\sigma_2 = 1.3 \times 10^{-2}$ nm^{-1}, respectively. By definition, σ_i values are calculated in units of nm^{-1} corresponding to real space length of $d_1 = 169$ nm and $d_2 = 238$ nm, with $d_k = \pi/\sigma_k$. Next, we analyze the decrements of the radial intensity profile $I(q_r)$. To this end, two sectors are defined ranging $\pm 10°$ around each axis with orientation γ_k. The signal is then transformed into polar coordinates, yielding the radial intensity distributions $I(q_r)\big|_{\gamma_k \pm 10°}$ along the principal direction. The signal is fitted to a power law decay

$$I(q_r) = a \times q_r^b + c. \tag{4}$$

Fit parameters are listed in Table 3.

TABLE 3 Fit Results following Eq. 4 Applied on Murine Myoblasts Depicted in Fig. 4

Axis	*a* ((photons × nm)/s)	*b* (1)	*c* (photons/s)	R^2 (1)	σ (1/nm)	*d* (nm)
1	2.0×10^{-4}	−3.95	2.7×10^{-2}	0.97	1.9×10^{-2}	169
2	1.2×10^{-4}	−3.74	3.1×10^{-2}	0.96	1.3×10^{-2}	238

Analysis of anisotropy in the diffraction patterns

To quantify the anisotropy of the SAXS patterns, we apply PCA to the diffraction patterns. PCA diagonalizes the covariance matrix

$$C = \begin{pmatrix} \mathrm{var}(q_y) & \mathrm{cov}(q_y, q_z) \\ \mathrm{cov}(q_z, q_y) & \mathrm{var}(q_z) \end{pmatrix} \tag{5}$$

with

$$\mathrm{cov}(q_i, q_j) = \frac{\sum_{m,n} I(m,n) \times [q_i(m,n) - \langle q_i \rangle] \times [q_j(m,n) - \langle q_j \rangle]}{\sum_{m,n} I(m,n)} \tag{6}$$

and the variances $\mathrm{var}(q_i) = \mathrm{cov}(q_i, q_i)$, $i = y, z$ as elements. $I(m, n)$ denotes the photon counts in pixel $m, n \in \mathbb{N}$ on the (masked) detector. The expectation value $\langle q_i \rangle$ for a single photon is given by

$$\langle q_i \rangle = \frac{\sum_{m,n} I(m,n) \times q_i(m,n)}{\sum_{m,n} I(m,n)}, \tag{7}$$

which in most experiments is the center of the primary beam. Solving the corresponding eigenvalue problem

$$C \times \vec{b}_k = \lambda_k \times \vec{b}_k \tag{8}$$

yields two eigenvectors $\vec{b}_k$, $k \in [1,2]$, which form a new orthogonal basis. The eigenvalues $\lambda_k = \sigma_k^2$ correspond to the variance in either direction. Both eigenvectors are scaled to unity ($\vec{e}_k = \vec{b}_k / \|\vec{b}_k\|$) and then sorted: the vector related to the largest eigenvalue describes the "line of best fit" (29,30), and is in the following denoted as the principal axis $\vec{e}_{\text{pa}}$, implying an orientation angle γ_{pa}. Because the cellular structure causing the signal is directed perpendicular to the diffraction pattern, the structure orientation angle θ_{pa} is defined by $\theta_{\text{pa}} = \gamma_{\text{pa}} + 90^\circ$. The anisotropy of the diffraction pattern is then defined locally for each scan point by the anisotropy parameter

$$\omega = \frac{|\lambda_1 - \lambda_2|}{\lambda_1 + \lambda_2}. \tag{9}$$

This results in an anisotropy map $\omega(y,z)$, which can be plotted along with the map of the orientation angle $\theta_{\text{pa}}(y,z)$, depicted as a white line, e.g., in Fig. 4 *b*.

PCA gives reliable results on SAXS patterns, as long as the empty beam is isotropic and the evaluated area is not interrupted by intermodular gaps of the detector. We thus restrict the evaluation of the diffraction signals to the inner module to avoid all gaps resulting in diffraction images as shown in Fig. 4 *c*. Before analysis, a ring-shape mask is applied setting values around the beamstop to zero. The eigenvectors are rescaled by the standard deviation $\sigma_k(y,z) = \sqrt{\lambda_k(y,z)}$

$$\vec{a}_k(y,z) = \sigma_k(y,z) \times \vec{e}_k(y,z), \tag{10}$$

illustrating the character of a signal: diffraction images with a pronounced anisotropic signal result in a relatively large aspect ratio $A_r(y,z) = |\vec{a}_{\text{pa}}(y,z)| / |\vec{a}_{k \neq \text{pa}}(y,z)|$ (Fig. 5 *a*). Contrarily, for isotropic signals, $A_r(y,z) \simeq 1$ (Fig. 5 *b*). Note that, by definition, $\vec{a}_k(y,z)$ values are calculated in units of nm^{-1}, supporting the representation of these vectors in reciprocal space.

Next, we consider cellular maps of entire cells and characterize them by defining global parameters to address cell type-specific structural differences. First, we measure orientational variations by calculating the 2D nematic order parameter s. The value s quantifies the variation of the principal axes' orientation within a cell. Following Liu et al. (31), we define the second rank tensor Q

$$Q_{\alpha,\beta} = \frac{1}{N_{\text{ROI}}} \sum_{y,z|_{\text{ROI}}} \left(2\, e_{\text{pa},\alpha}(y,z) e_{\text{pa},\beta}(y,z) - \delta_{\alpha,\beta}\right), \tag{11}$$

with $\alpha,\beta = 1,2$, the total number of diffraction patterns N_{ROI} within the region of interest and the principal axis defined by

$$\vec{e}_{\text{pa}}(y,z) = \begin{pmatrix} \cos(\theta_{\text{pa}}(y,z)) \\ \sin(\theta_{\text{pa}}(y,z)) \end{pmatrix}. \tag{12}$$

Insertion of Eq. 12 in Eq. 11 and solving the eigenvalue problem of Q then leads to an expression for the order parameter s, being the positive eigenvalue

$$s = \frac{1}{N_{\text{ROI}}} \sqrt{\left(\sum_{y,z|_{\text{ROI}}} \sin(2\,\theta_{\text{pa}}(y,z))\right)^2 + \left(\sum_{y,z|_{\text{ROI}}} \cos(2\,\theta_{\text{pa}}(y,z))\right)^2} \tag{13}$$

(for details, see Section S10.5 in the Supporting Material). The corresponding eigenvector is the director of the evaluated area; see the black arrow in Fig. 6 *c*. In addition, an overall anisotropic scattering strength of the cell can be quantified by averaging the local anisotropy parameters $\omega(y,z)$

$$\Omega = \frac{1}{N_{\text{ROI}}} \sum_{y,z|_{\text{ROI}}} \omega(y,z). \tag{14}$$

Fig. 6 shows a freeze-dried mi-hMSC, along with Fig. 6 *a*, the corresponding OAV-image and Fig. 6 *b*, the x-ray dark field map. A region of interest as marked in pink is evaluated

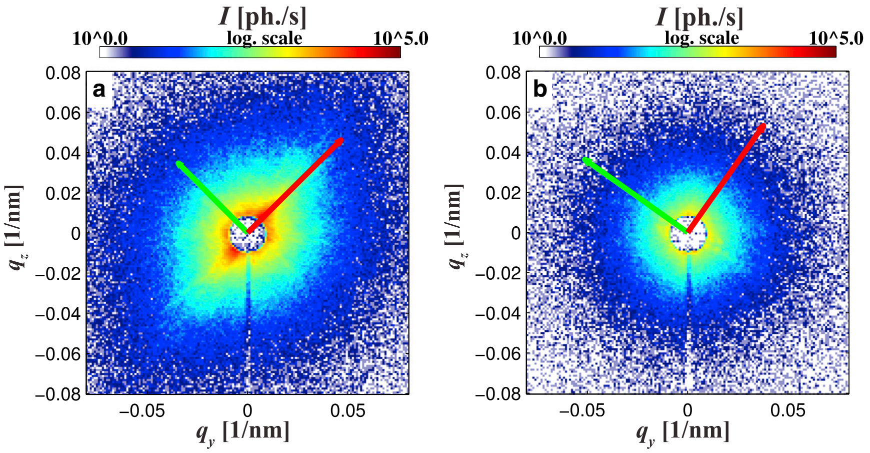

FIGURE 5 Examples for (*a*) anisotropic and (*b*) isotropic diffraction patterns are shown along with the resulting principal axis of the PCA. Vectors are rescaled by the standard deviation $\sigma_k(y,z)$ as denoted in Eq. 10. For presentation purposes, a common scaling factor is introduced for the orientation vectors, keeping the aspect ratio unaffected. To see this figure in color, go online.

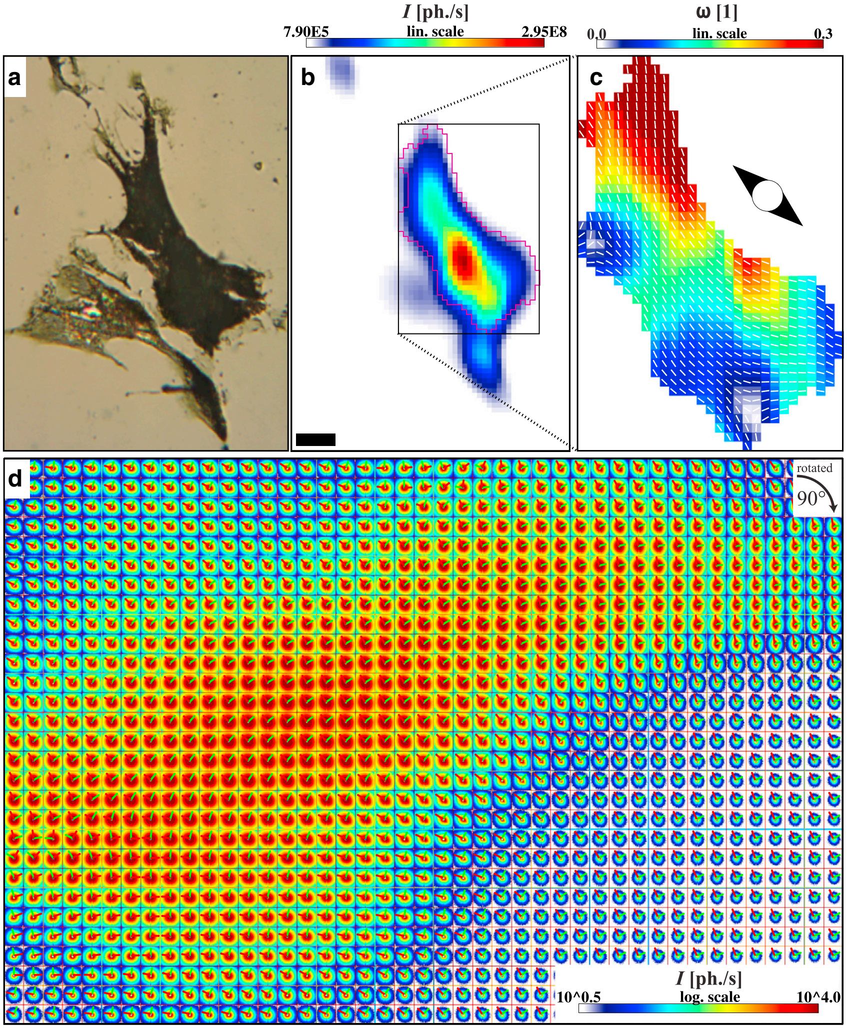

FIGURE 6 (*a*) OAV-image of lyophilized muscle-induced hMSCs before recording. (*b*) X-ray dark field scan recorded with the micro-SAXS setup. Scale bar, 40 μm. (*c*) PCA of the region marked in (*b*). All contributions outside this area are set to zero. (*d*) Composite image of the scan area shown in (*c*), dataset rotated clockwise by 90°. By PCA, two basis vectors are computed for every diffraction image. To see this figure in color, go online.

by PCA and shown in Fig. 6 *c*; all contributions outside this region are set to zero. PCA results can then be depicted in a composite image, again showing the two rescaled eigenvectors $\vec{a}_k(y,z)$ at each scan position in Fig. 6 *d*. This dataset leads to values of $s = 0.65$ and $\Omega = 0.15$. Further examples are shown in the Supporting Material (Section S10.5 in the Supporting Material).

Comparison of different cell types

To compare different cell types in view of the structural observables defined above, the results of multiple scans performed on freeze-dried naive and mi-hMSCs, as well as murine myo- (C2C12) and fibroblasts (NIH-3T3) are compiled in this section (Fig. 7). For analysis, the x-ray dark field image of every scan was masked separating the diffraction data into areas of relatively strong and weak scattering signals. Comparison of dark field and OAV-image then leads to an adjusted mask, defining an adequate region to calculate these observables for single cells as detailed in Section S10.5 in the Supporting Material. Fig. 7 *a* shows the results for the mean scattering intensity $\langle I \rangle_{\mathrm{cell}}$ for single cells and different cell types:

$$\langle I \rangle_{\mathrm{cell}} = \frac{1}{N_{\mathrm{ROI}}} \sum_{y,z|_{\mathrm{ROI}}} I_{\mathrm{diffraction}}(y,z). \qquad (15)$$

The mean for each cell type is depicted as bars (and the standard deviation as error bars) showing an increase in scattering intensity from naive to mi-hMCSs and from murine myo- to fibroblasts.

Because contributions to $\langle I \rangle_{\mathrm{cell}}$ can originate from both isotropic and anisotropic portions of the signal, data are further analyzed in this respect by the determination of Ω for every cell following Eq. 14 (Fig. 7 *b*). When comparing naive hMSCs to all other cell types, a shift in data is apparent, supporting our hypothesis of the former being rather unstructured.

Addressing orientational variations, we then calculate the 2D nematic order parameter *s*, leading to relatively high values for all murine cells (Fig. 7 *c*). In fact, regarding the

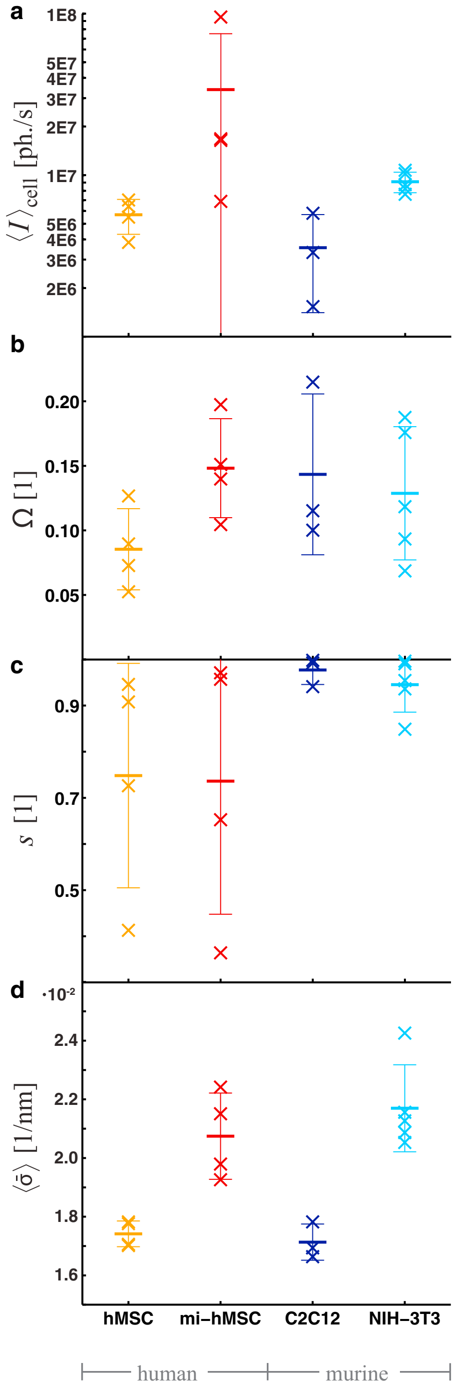

FIGURE 7 Statistical evaluation of the mean (*a*) scattering intensity, (*b*) anisotropy parameter Ω (Eq. 14), (*c*) 2D nematic order parameter *s* (Eq. 13), and (*d*) averaged standard deviation of diffraction patterns $\overline{\sigma}$ for different cell types. To see this figure in color, go online.

local orientation angles $\theta(y,z)$ of murine cells, a strict overall orientation is apparent (see also Section S10.5 in the Supporting Material).

Furthermore, we quantify the radial intensity profile $I(q_r)$ by determining the mean standard deviation $\langle\overline{\sigma}\rangle_{cell}$, which is a convenient and straightforward measure of a representative scale of Fourier components, independent of any particular model or fitting ansatz with

$$\langle\overline{\sigma}\rangle_{cell} = \frac{\sum_{y,z|_{ROI}} \overline{\sigma}(y,z)}{N_{ROI}} = \frac{\sum_{y,z|_{ROI}} \sqrt{\frac{\lambda_1(y,z)+\lambda_2(y,z)}{2}}}{N_{ROI}} \tag{16}$$

(Fig. 7 *d*). Relatively large values for $\langle\overline{\sigma}\rangle_{cell}$ are obtained in case of mi-hMSCs and murine fibroblasts implying significant scattering for high q values caused by small, ordered structures within the cells.

DISCUSSION

Understanding cellular processes in general, and stem cell differentiation in particular, requires advanced biophysical methods to unravel the evolution of subcellular structures. Here we apply scanning small-angle x-ray scattering with focused beams to biological cells to probe the native electron density distribution. While the full potential of these methods will probably only unfold once suitable modeling of the local and highly anisotropic diffraction patterns becomes available, we here adopt a strategy of automatized empirical analysis of the diffraction data. In particular, we show that PCA is suited to track down local orientation angles and the degree of anisotropy under the precondition of a sufficiently isotropic direct beam. From PCA results, a global nematic order parameter can be derived in a straightforward manner. In particular, we have introduced four structural parameters, which can be computed from the scanning SAXS data in a model-free approach: the mean scattering intensity $\langle I\rangle_{cell}$; the mean anisotropy parameter Ω; the 2D nematic order parameter *s*; and the mean variance of the momentum transfer $\langle\overline{\sigma}\rangle$, corresponding to typical length scales dominating the diffraction. This approach provides surprising insight into the evolution of local structures and anisotropy of the cytoskeleton. While we cannot trace down the individual molecular components producing the diffraction, we can record and analyze the locally dominating Fourier components up to near-molecular scales with a real-space resolution, which is still high enough to distinguish between different parts of the cell, such as nucleus or cytoplasm. The results support the hypothesis that naive hMSCs are comparatively void of structure, lacking the pronounced Fourier components and anisotropies observed in the diffraction patterns of other cell types. Apart from these first results, which require further work and extension, the primary goal of this work was to further establish cellular micro- and nano-SAXS as a novel label-free imaging technique, enhanced by technical developments including optical setups (recent publications demonstrated beam focusing in the nanometer-range (32,33)), sample environments such as highly transmissive chambers (Silson, Northampton, UK), and the analysis tools described here. Altogether, diffraction data from biological cells is recorded at a signal/noise that was believed to be

impossible just a few years ago. Importantly, we showed that cryogenic conditions can suppress beam damage and preserve the structure of the specimen allowing multiple scans or even the combination of different recording methods (e.g., nano-SAXS and ptychography (19,34) or nano-SAXS and holography (23)) on the same area. At the same time, it becomes clear that hydrated cells are much more challenging, concerning firstly suitable x-ray compatible cell culture chambers, and secondly the achievable signal/noise. To this end, future work needs to address further optimization, in particular in view of suitable window materials, with high transmission and low background in combination with good adhesion probabilities for the cells of interest. Reduction in channel depth, photon energy, and better background subtraction also need to be addressed, as well as possible mechanisms to reduce radiation damage in the room temperature setting, for example by constant flow, free radical scavengers, and measurement protocols.

SUPPORTING MATERIAL

Supporting Materials and Methods, eleven figures, and one table are available at http://www.biophysj.org/biophysj/supplemental/S0006-3495(15)04759-1.

AUTHOR CONTRIBUTIONS

T.S. and F.R. designed research; M.B. prepared samples; M.B. and C.W. established sample preparation workflow; A.D., T.S., and M.B. conceived and implemented the beamline setup; M.B., M.P., T.S., F.R., and M.O. prepared the beamtime and recorded data; M.B. analyzed data; T.S., F.R., M.P., C.W., and M.O. gave constant scientific feedback; and M.B., T.S., M.P., and F.R. wrote the manuscript.

ACKNOWLEDGMENTS

We thank Dr. Robin Wilke, Dr. Michael Sprung, and Yihui Xu for their support during beamtimes as well as Prof. Dr. Sarah Köster, Dr. Britta Weihausen, and Johannes Hagemann for many fruitful discussions concerning SAXS experiments. We thank Florian Schlosser, Galina Kudryasheva, and Susanne Karsch for advice in handling NIH-3T3-, C2C12-, and the muscle induction of human mesenchymal stem cells. Moreover, we thank Jochen Herbst, Bastian Hartmann, and Mike Kanbach for their support concerning substrate preparation. Finally, we thank the P10-staff at the Deutsches Elektronen-Synchrotron (Hamburg, Germany) and the cSAXS-staff at the Paul Scherrer Institut (Villigen, Switzerland) for making these x-ray recordings possible.

This work was funded by the Deutsche Forschungsgemeinschaft via the Sonderforschungsbereich No. SFB 755 "Nanoscale Photonic Imaging" within projects No. C01 and No. B08, the Sonderforschungsbereich No. SFB 937 "Collective Behavior of Soft and Biological Matter" within project No. A11, the Virtual Institute No. VH-VI-403 "In Situ Nano Imaging of Biological and Chemical Processes" (Helmholtz Society), and the Volkswagen Stiftung within the Niedersachsen Israel framework (grant No. MWK-VWZN2722).

REFERENCES

1. Pittenger, M. F., A. M. Mackay, ..., D. R. Marshak. 1999. Multilineage potential of adult human mesenchymal stem cells. *Science.* 284:143–147.
2. Singer, N. G., and A. I. Caplan. 2011. Mesenchymal stem cells: mechanisms of inflammation. *Annu. Rev. Pathol.* 6:457–478.
3. Zuk, P. A., M. Zhu, ..., M. H. Hedrick. 2002. Human adipose tissue is a source of multipotent stem cells. *Mol. Biol. Cell.* 13:4279–4295.
4. Zemel, A., F. Rehfeldt, ..., S. A. Safran. 2010. Optimal matrix rigidity for stress fiber polarization in stem cells. *Nat. Phys.* 6:468–473.
5. Rehfeldt, F., A. E. Brown, ..., D. E. Discher. 2012. Hyaluronic acid matrices show matrix stiffness in 2D and 3D dictates cytoskeletal order and myosin-II phosphorylation within stem cells. *Integr. Biol. (Camb).* 4:422–430.
6. Engler, A. J., S. Sen, ..., D. E. Discher. 2006. Matrix elasticity directs stem cell lineage specification. *Cell.* 126:677–689.
7. Paluch, E. K., C. M. Nelson, ..., W. Federle. 2015. Mechanotransduction: use the force(s). *BMC Biol.* 13:47.
8. Wei, X., X. Yang, ..., Y. F. Shi. 2013. Mesenchymal stem cells: a new trend for cell therapy. *Acta Pharmacol. Sin.* 34:747–754.
9. Ullah, I., R. B. Subbarao, and G. J. Rho. 2015. Human mesenchymal stem cells - current trends and future prospective. *Biosci. Rep.* 35: e00191.
10. Klar, T. A., S. Jakobs, ..., S. W. Hell. 2000. Fluorescence microscopy with diffraction resolution barrier broken by stimulated emission. *Proc. Natl. Acad. Sci. USA.* 97:8206–8210.
11. Westphal, V., and S. W. Hell. 2005. Nanoscale resolution in the focal plane of an optical microscope. *Phys. Rev. Lett.* 94:143903.
12. Hess, S. T., T. P. Girirajan, and M. D. Mason. 2006. Ultra-high resolution imaging by fluorescence photoactivation localization microscopy. *Biophys. J.* 91:4258–4272.
13. Betzig, E., G. H. Patterson, ..., H. F. Hess. 2006. Imaging intracellular fluorescent proteins at nanometer resolution. *Science.* 313:1642–1645.
14. Rust, M. J., M. Bates, and X. Zhuang. 2006. Sub-diffraction-limit imaging by stochastic optical reconstruction microscopy (STORM). *Nat. Methods.* 3:793–795.
15. Svergun, D. I., and M. H. J. Koch. 2003. Small-angle scattering studies of biological macromolecules in solution. *Rep. Prog. Phys.* 66:1735–1782.
16. Koch, M. H., P. Vachette, and D. I. Svergun. 2003. Small-angle scattering: a view on the properties, structures and structural changes of biological macromolecules in solution. *Q. Rev. Biophys.* 36:147–227.
17. Putnam, C. D., M. Hammel, ..., J. A. Tainer. 2007. X-ray solution scattering (SAXS) combined with crystallography and computation: defining accurate macromolecular structures, conformations and assemblies in solution. *Q. Rev. Biophys.* 40:191–285.
18. Claessens, M. M., C. Semmrich, ..., A. R. Bausch. 2008. Helical twist controls the thickness of F-actin bundles. *Proc. Natl. Acad. Sci. USA.* 105:8819–8822.
19. Wilke, R. N., M. Priebe, ..., T. Salditt. 2012. Hard x-ray imaging of bacterial cells: nano-diffraction and ptychographic reconstruction. *Opt. Express.* 20:19232–19254.
20. Weinhausen, B., J.-F. Nolting, ..., S. Köster. 2012. X-ray nano-diffraction on cytoskeletal networks. *New J. Phys.* 14:085013.
21. Weinhausen, B., O. Saldanha, ..., S. Köster. 2014. Scanning x-ray nanodiffraction on living eukaryotic cells in microfluidic environments. *Phys. Rev. Lett.* 112:088102.
22. Priebe, M., M. Bernhardt, ..., T. Salditt. 2014. Scanning x-ray nanodiffraction on *Dictyostelium discoideum*. *Biophys. J.* 107:2662–2673.
23. Wilke, R. N., M. Hoppert, ..., T. Salditt. 2015. Quantitative x-ray phase contrast waveguide imaging of bacterial endospores. *J. Appl. Cryst.* 48:464–476.
24. Kalbfleisch, S., H. Neubauer, ..., T. Salditt. 2011. The Göttingen Holography Endstation of Beamline P10 at PETRA III/DESY. *AIP Conf. Proc.* 1365:96–99.
25. Takahashi, Y., A. Suzuki, ..., M. Nakasako. 2013. Coherent diffraction imaging analysis of shape-controlled nanoparticles with focused hard x-ray free-electron laser pulses. *Nano Lett.* 13:6028–6032.

26. Henrich, B., A. Bergamaschi, …, B. Schmitt. 2009. Pilatus: a single photon counting pixel detector for x-ray applications. *In* Nuclear Instruments and Methods in Physics Research Section A: Accelerators, Spectrometers, Detectors and Associated Equipment. 607:247–249.

27. Howells, M. R., T. Beetz, …, D. Starodub. 2009. An assessment of the resolution limitation due to radiation-damage in x-ray diffraction microscopy. *J. Electron Spectrosc. Relat. Phenom.* 170:4–12, (Radiation Damage.).

28. Meisburger, S. P., M. Warkentin, …, R. E. Thorne. 2013. Breaking the radiation damage limit with Cryo-SAXS. *Biophys. J.* 104:227–236.

29. Pearson, K. 1901. On lines and planes of closest fit to systems of points in space. *Philos. Mag.* 2:559–572.

30. Jackson, J. E. 1991. A User's Guide to Principal Components. Wiley, New York.

31. Liu, B., T. H. Besseling, …, A. van Blaaderen. 2014. Switching plastic crystals of colloidal rods with electric fields. *Nat. Commun.* 5:3092.

32. Mimura, H., S. Handa, …, K. Yamauchi. 2010. Breaking the 10 nm barrier in hard-x-ray focusing. *Nat. Phys.* 6:122–125.

33. Osterhoff, M., C. Eberl, …, T. Salditt. 2015. Towards multi-order hard x-ray imaging with multilayer zone plates. *J. Appl. Cryst.* 48:116–124.

34. Piazza, V., B. Weinhausen, …, S. Köster. 2014. Revealing the structure of stereociliary actin by x-ray nanoimaging. *ACS Nano.* 8:12228–12237.

Biophysical Journal

Supporting Material

X-Ray Micro- and Nanodiffraction Imaging on Human Mesenchymal Stem Cells and Differentiated Cells

Marten Bernhardt,[1] Marius Priebe,[1] Markus Osterhoff,[1] Carina Wollnik,[2] Ana Diaz,[3] Tim Salditt,[1,*] and Florian Rehfeldt[2,*]

[1]Institut für Röntgenphysik, Georg-August-Universität Göttingen, Göttingen, Germany; [2]Drittes Physikalisches Institut - Biophysik, Georg-August-Universität Göttingen, Göttingen, Germany; and [3]Paul Scherrer Institut, Villigen, Switzerland

*Correspondence: tsaldit@gwdg.de; florian.rehfeldt@phys.uni-goettingen.de

10.1 X-ray Micro- and Nanodiffraction Setup at cSAXS (PSI) & P10 (PETRAIII)

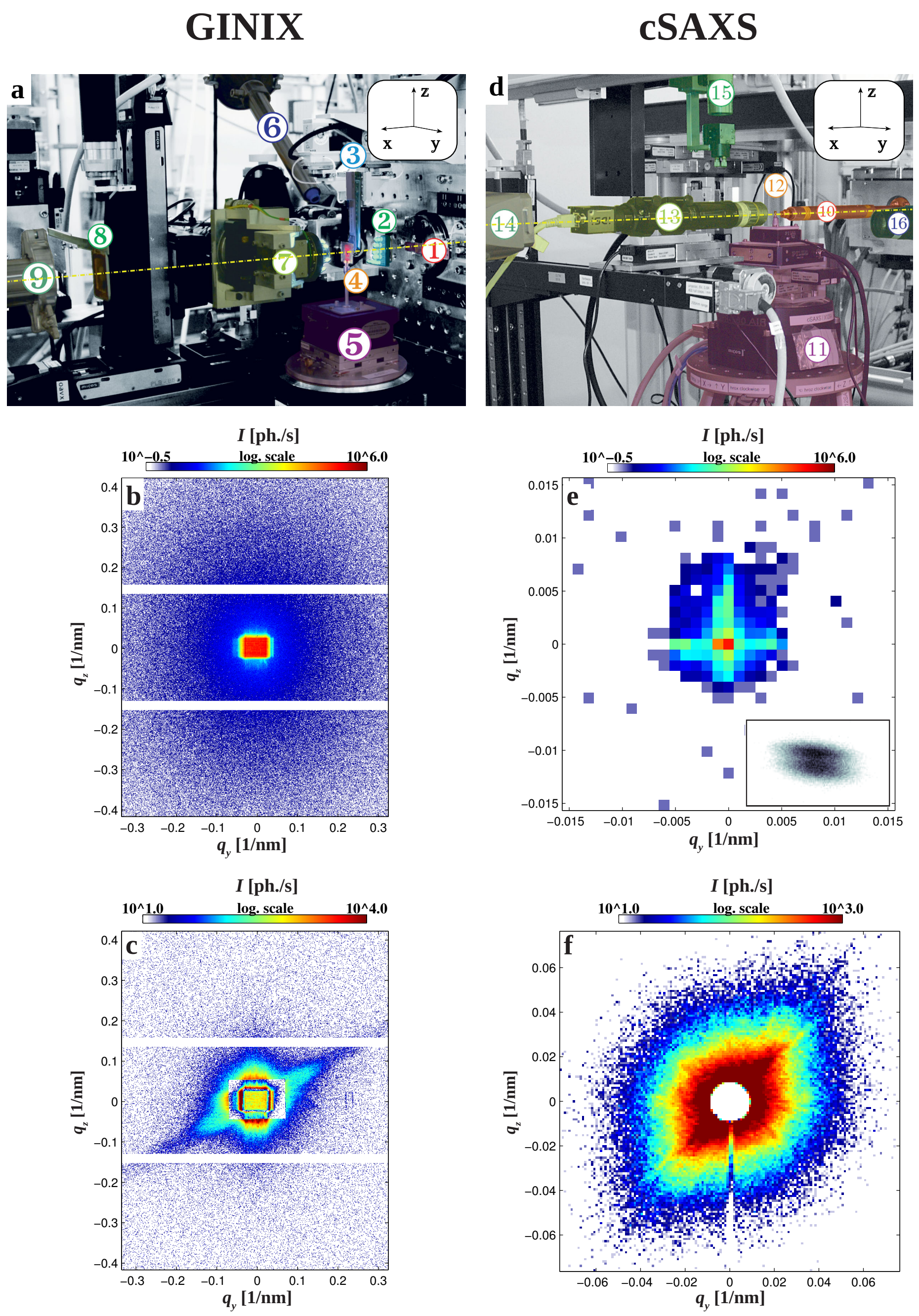

Figure 1: *left:* (*a*) Photograph of the nano-focus setup GINIX (4, 7) together with far field images of (*b*) the direct beam and (*c*) a representative cellular diffraction signal recorded at GINIX. *right:* (*d*) Photograph of the micro-focus setup together with far field images of (*e*) the direct beam and (*f*) a representative cellular diffraction signal recorded at cSAXS (1). The beam profile in focus could be visualized by a scintillator, see *e* (inset).

Fig. 1 *a* shows a photograph of the Göttingen Instrument for Nano-Imaging with x-rays (GINIX) (4) at the P10-beamline of DESY's PETRAIII storage ring: A set

of Kirkpatrick-Baez mirrors (not shown) enabled beam focussing to a size of about 200 nm. The beam left the evacuated tube at (1) impinging the sample (here: a self-assembled glass-chamber, (4)) about 20 cm downstream from the mirrors. Two soft-edge apertures (3) block parasitic scattering. The sample was mounted on a motorized stage (5) enabling sample translation with respect to the beam. An on-axis video-microscope (OAV, Optique Peter, France) (7) with LED illumination (2) was used for orientation on the substrate. Optionally, cryogenic conditions (6) could be applied (Cryostream, Oxford instruments, UK). Diffraction patterns were recorded in ≈ 5.1 m distance by the single photon counting pixelated detector Pilatus 300k (Dectris, Switzerland). An evacuated tube (9) minimized absorption. The primary beam could be blocked by multiple semi-transparent beamstops placed either in front of the tube (8) or inside (not shown). The focused photon flux was $I_0 = 1.29 \cdot 10^{11}$ ph./s as determined by direct beam recordings, see *b*. A representative cellular diffraction image is shown in *c*.
Fig. 1 *d* shows a photograph of the micro-SAXS data at cSAXS-beamline of the Swiss Light Source at the Paul Scherrer Institut in Villigen, Switzerland. The x-ray beam was focused by a Si(111) crystal monochromator in horizontal and a Rh-coated mirror in vertical direction enabling a spotsize of about 40 µm at the sample position. The beam left the vacuum tube through a 4 µm-thick mica-window at (10) impinging the sample (12) about 5 m downstream from the mirrors. Multiple step motors and a hexapod (11) enabled the translation of the sample with respect to the beam. An on-axis optical microscope (13) and a camera (16) were used for orientation on the substrate and the surveillance of the sample. The shape and size of the beam at the sample position was determined by a scintillator based x-ray microscope (15). An evacuated tube (14) of 7 m length spans the distance from the sample to the single photon counting pixelated detector Pilatus 2M (3). For x-ray measurements the on-axis microscope was retracted and the flight tube was approached to the specimen. The focused photon flux was $I_0 = 1.44 \cdot 10^{11}$ ph./s as determined by direct beam recordings with filters, while the setup provides an extraordinarily clean direct beam signal, see *e*. A representative cellular diffraction image is shown in *f*.

10.2 Sample preparation: Cells

The cells used were obtained from the following sources:
NIH-3T3s (murine embryonic fibroblasts): Deutsche Sammlung von Mikroorganismen und Zellkulturen (DSMZ), DSMZ-no. ACC 59; **hMSC**s (human mesenchymal stem cells): Lonza, Switzerland, cat.-no. PT-2501; **C2C12** (murine myoblasts): DSMZ, DSMZ-no. ACC 565.
Cells were incubated at 37 °C at 5 % CO_2 in vent-cap culture flasks and on the day of splitting were washed at least once with Dulbecco's phosphate-buffered saline (DPBS) before trypsinization. Splitting was done every 4th day at most. Muscle-induced hMSCs (mi-hMSCs) were derived from naive hMSCs and remained attached up to 10 days. The medium was changed to muscle induction medium (MIM) as reported by Engler *et al.* (2, SM), more than a week before fixation.

10.3 Cryogenic protection of freeze-dried samples

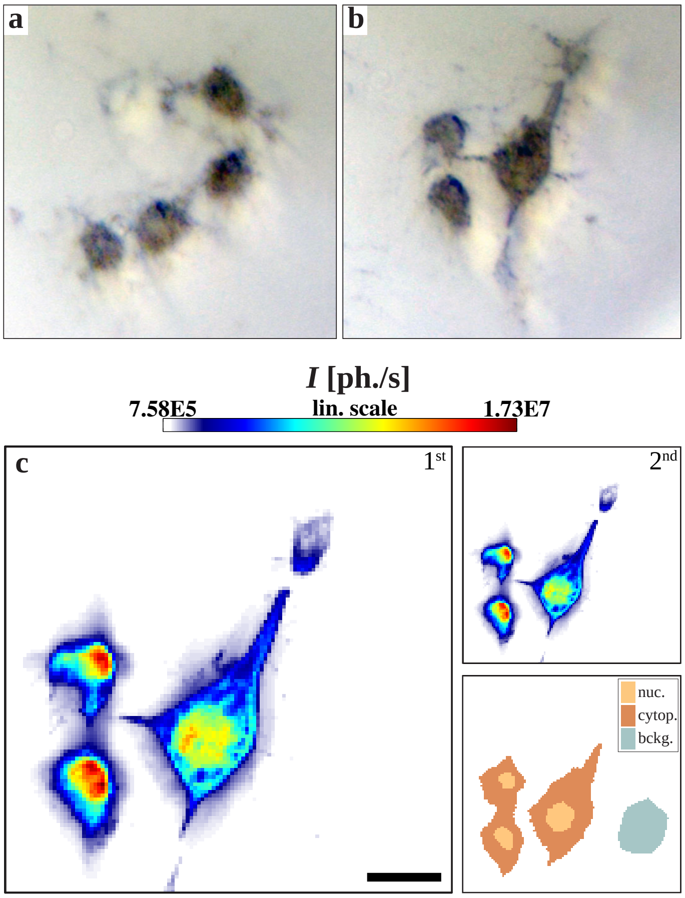

Figure 2: Optical micrographs of the scan areas (*a*) “5 cell”- and (*b*) “3 cell”-arrangement treated in Sec. 3 (main article). (*c*) First and second dark-field map of the “3 cell”-arrangement together with the stxm mask of the second scan series. Scalebar: 20 µm.

Towards establishing cryoprotection as a tool to preserve the diffraction signal of freeze-dried samples, two scan series were performed on adherent murine fibroblasts comprising three and two successive scans, respectively. Fig. 2 *a-b* show the OAV-images of the respective scan areas. *c* In agreement with the first scan series, x-ray dark-field images of the second scan series indicate no significant loss of scattering signal. In order to investigate the radial intensity profile $I(q_r)$, the corresponding first scan of both series was masked (see lower right corner) separating nucleic-, cytoplasmic- and background-regions. This mask was then transferred to all following scans adjusting translational alterations and differing stepsizes. After calculating an averaged 2D image for the nucleus-, cytoplasm- and background of each scan, data were transformed and substracted obtaining the background-corrected radial intensity profile for each scan, see Fig. 3 *a-b*. Data were then fitted by a power law function:

$$I(q_r) = a \cdot q_r^b + c \; . \tag{1}$$

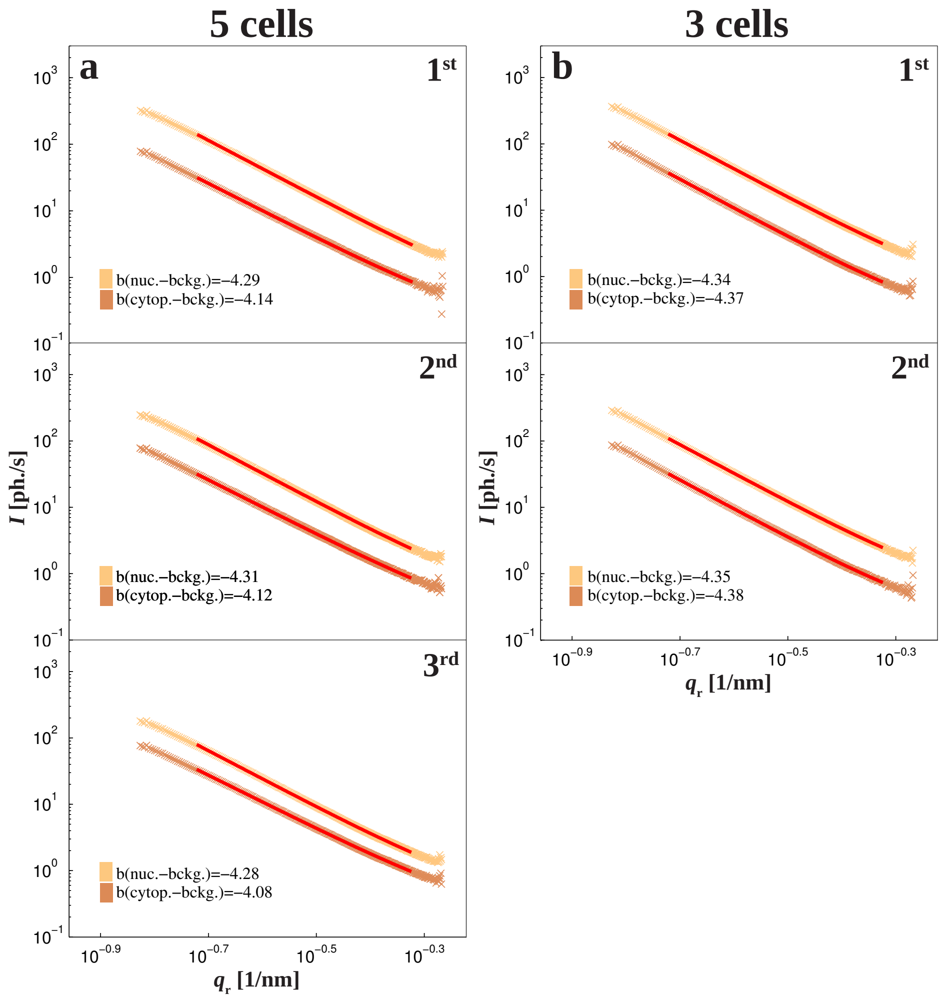

Figure 3: (*a*-*b*) After calculating a 2D-average signal, the respective background-corrected radial intensity profiles $I(q_\mathrm{r})$ were determined for each scan and fitted to a power law function fitted to the background-corrected signal of the nucleus (upper curve) and the cytoplasm (lower curve), respectively.

10.4 Sample Environment and Chambers

Wet sample experiments

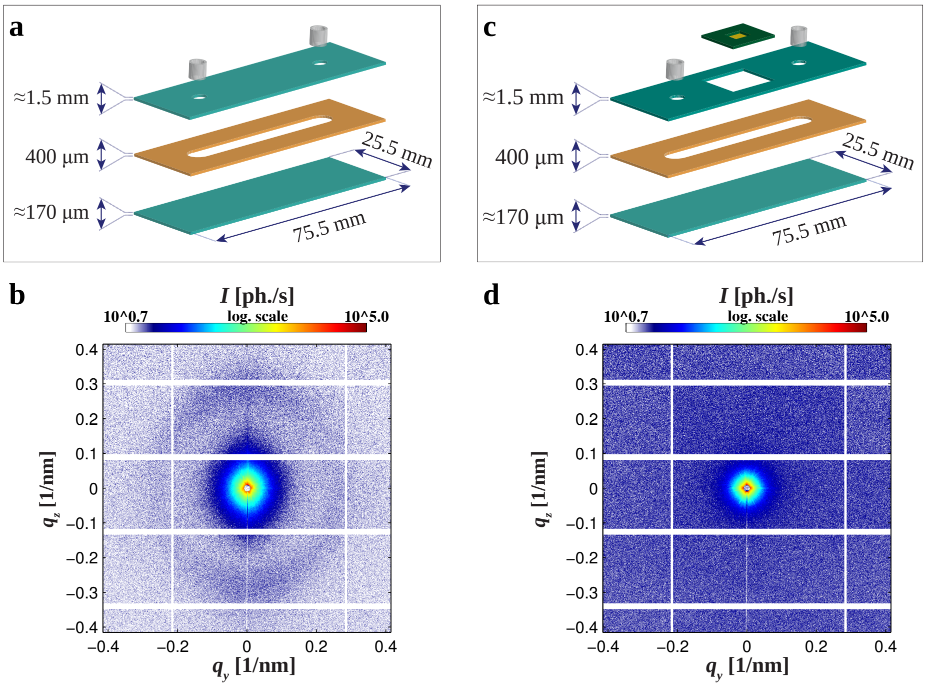

Figure 4: *left:* (*a*) Sketch of a commercial chamber (ibidi, Germany). (*b*) The diffraction signal of an equivalent 6-channel geometry sample (ibitreat, channel height: 400 µm, 6-channel cut) reveals a ring related to the polymer material of the upper slide. *right:* (*c*-*d*) When replacing the upper slide by highly transmissive Si_3N_4-windows this signal has vanished (ibitreat, channel height: 400 µm, 1-channel cut).

The development of sample environments that are simultaneously compatible with cell culture and x-ray microscopy is challenging. Previous approaches have used custom-built PMMA based microfluidic chambers in combination with ultrathin Si_3N_4-foils as x-ray windows onto which cells could adhere (8). Slides with microfluidic channels are commercially available for visible light microscopy (e. g., ibidi, Germany) and provide various biochemical coatings and physical surface treatments as well as sufficient gas exchange for the use of carbonate buffer (sketched in Fig. 4 *a*). We have tested these slides for the present x-ray nano-SAXS applications. Transmission at photon energy of $E_{\mathrm{ph.}} = 13.8\,\mathrm{keV}$ was considered satisfactory, and even at $E_{\mathrm{ph.}} = 8.7\,\mathrm{keV}$ acceptable, but the background of the SAXS patterns was found to be too high, despite the fact that the main polymer used is of low residual signal compared to other materials at similar thickness (5). In particular, our recordings reveal a characteristic peak at $q_r \approx 0.27\,\mathrm{nm}^{-1}$ (shown in *b*) caused by the upper slide of the polymer channel with an estimated thickness of about 1.5 mm. Together with the manufacturer we have developed an x-ray compatible design that includes an x-ray transparent and scattering free Si_3N_4-window (Silson, UK), replacing parts of the upper slide of the polymer channel (Fig. 4 *c*-*d*). Another chamber type with a sealed volume consists of two coverslips of borosilicate glass with an average thickness of ≈100 µm as window material and a punched piece of Parafilm serving as a spacer (not shown).

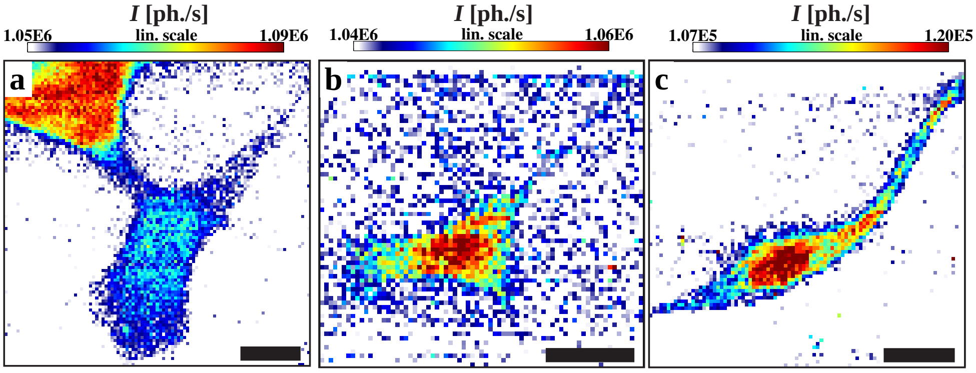

Figure 5: (*a-b*) X-ray dark-field images of murine myoblasts recorded in a commercially available chamber (ibidi, Germany). Scalebars: 10 µm and 20 µm. (*c*) Naive hMSC mounted on borosilicate glass and placed in an improvised glass-chamber. Scalebar: 20 µm.

Figure 5 shows nano-SAXS results obtained on hydrated fixed cells: In *a* & *b* the corresponding x-ray dark-field images are shown for murine myoblasts, cultivated in commercial cell culture slides (channel height: 200 µm, collagen IV coated bottom layer) and recorded at a photon energy of $E_{\mathrm{ph.}}$ =13.8 keV. Despite the elevated background and the reduced signal due to a smaller contrast between cells and solution, the cells can be clearly identified in the dark-field images. In *c*, the x-ray dark-field image is shown for a naive hMSC in a home built glass coverslip chamber, recorded at a photon energy of $E_{\mathrm{ph.}}$ =7.9 keV. The nucleus can be distinguished by eye, but the signal-to-noise ratio is too low to perform quantitative analysis on the diffraction data. For such low signals, even the slight intensity variations due to the top-up mode of the storage ring, i. e. the periodic refill of electrons, become noticable and must be corrected (not shown).

Frozen-hydrated samples

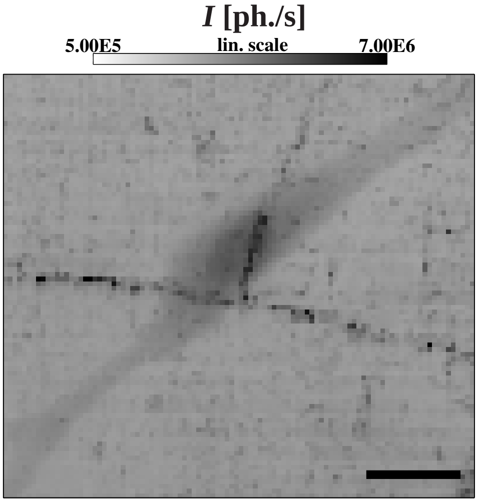

Figure 6: X-ray dark-field image of a frozen-hydrated naive hMSC. Scalebar: 20µm.

GINIX provides a cryogenic sample environment. Thus, one can avoid the invasive last step of freeze-drying eukariotic cells leaving them in a frozen-hydrated state.

Fig. 6 shows the x-ray dark-field image of a frozen-hydrated naive hMSC. The nucleus can be clearly distinguished from the cytoplasm. Two cracks show a strong overall scattering signal with pronounced anisotropy.

10.5 Structural differences among cell lines

Applying x-ray micro-diffraction on freeze-dried samples enables the quantification of local structures within a cell and a structural comparison among different cell lines. In this work, we propose ω obtained by PCA as a local and its respective mean Ω as a global order parameter describing anisotropy. Ω measures the overall anisotropic character, leading to relatively high values in case of a strong occurrence of parallel structures inside a cell. Data gathered so far indicate structural differences among cell lines. For a more detailed view, one representative scan for each cell line is chosen: See Fig. 7 for naive human mesenchymal stem cells, Fig. 6 (main article) for muscle-induced hMSCs, Fig. 8 for murine myoblasts and Fig. 9 for murine fibroblasts. All figures show the on-axis microscopic image (OAV) of the cell in *a* and the x-ray dark-field in *b*. After defining a region of interest, indicated by a black frame, a STXM mask derived from the dark-field image is applied on the corresponding PCA results, see *c*, as indicated by a pink contour line in *b*. The composite image is shown in *d* including all principal axes of the respective diffraction images.
In view of orientational variations within a cell, quantified by the nematic order parameter *s*, data reveal relatively low values for naive and muscle-induced hMSCs and relatively high values for murine myo- and fibroblasts. This finding becomes apparent when reviewing the PCA results of the representatives chosen. Fig. 10 shows the distribution of structure orientation angles θ_{pa} in a histogram. θ_{pa} covers a broad range of angles in case of mesenchymal stem cells while the distibution is narrow in the case of murine myo- and fibroblasts.

According to Liu *et al.* (6) *s* is derived by the second rank tensor *Q* as follows:

$$Q_{\alpha\beta} = \frac{1}{N_{\text{ROI}}} \sum_{y,z|_{\text{ROI}}} \left(2\, e_{\text{pa},\alpha}(y,z)\, e_{\text{pa},\beta}(y,z) - \delta_{\alpha\beta}\right)$$

$$Q = \frac{2}{N_{\text{ROI}}} \cdot \begin{pmatrix} \sum\limits_{y,z|_{\text{ROI}}} \left(e_{\text{pa},1}(y,z) e_{\text{pa},1}(y,z) - \frac{1}{2}\right) & \sum\limits_{y,z|_{\text{ROI}}} e_{\text{pa},1}(y,z) e_{\text{pa},2}(y,z) \\ \sum\limits_{y,z|_{\text{ROI}}} e_{\text{pa},2}(y,z) e_{\text{pa},1}(y,z) & \sum\limits_{y,z|_{\text{ROI}}} \left(e_{\text{pa},2}(y,z) e_{\text{pa},2}(y,z) - \frac{1}{2}\right) \end{pmatrix}$$

$$\stackrel{*}{=} \frac{2}{N} \cdot \begin{pmatrix} \sum\limits_{y,z|_{\text{ROI}}} \left((\cos(\theta_{\text{pa}}(y,z)))^2 - \frac{1}{2}\right) & \sum\limits_{y,z|_{\text{ROI}}} \cos(\theta_{\text{pa}}(y,z)) \sin(\theta_{\text{pa}}(y,z)) \\ \sum\limits_{y,z|_{\text{ROI}}} \sin(\theta_{\text{pa}}(y,z)) \cos(\theta_{\text{pa}}(y,z)) & \sum\limits_{y,z|_{\text{ROI}}} \left((\sin(\theta_{\text{pa}}(y,z)))^2 - \frac{1}{2}\right) \end{pmatrix}$$

* assuming a general vector $\vec{e}_{\text{pa}}(y,z) = \begin{pmatrix} e_1(y,z) \\ e_2(y,z) \end{pmatrix} = \begin{pmatrix} \cos(\theta_{\text{pa}}(y,z)) \\ \sin(\theta_{\text{pa}}(y,z)) \end{pmatrix}$ showing along the principal axis.

We then determined the eigenvalues $s_{1,2}$ of the equation:

$$Q \cdot \vec{b} = s \cdot \vec{b}$$

using Wolfram Mathematica 8 (Wolfram Research, IL, USA). The nematic order parameter is the positive eigenvalue $s_{1\vee 2} > 0$.

$$s_{1\vee 2} = \frac{1}{2N_{\text{ROI}}} \left(-2N_{\text{ROI}} + \sum_{y,z|_{\text{ROI}}} 2(\cos(\theta_{\text{pa}}(y,z)))^2 \right.$$
$$+ \sqrt{4 \left(\sum_{y,z|_{\text{ROI}}} 2\cos(\theta_{\text{pa}}(y,z)) \sin(\theta_{\text{pa}}(y,z)) \right)^2 + \left(\sum_{y,z|_{\text{ROI}}} 2\left(\cos(\theta_{\text{pa}}(y,z))\right)^2 - \sum_{y,z|_{\text{ROI}}} 2\left(\sin(\theta_{\text{pa}}(y,z))\right)^2 \right)^2}$$
$$\left. + \sum_{y,z|_{\text{ROI}}} 2\left(\sin(\theta_{\text{pa}}(y,z))\right)^2 \right)$$

$$
\begin{aligned}
&\overset{(1)}{=} \frac{1}{2N_{\mathrm{ROI}}} \left(\sqrt{4\left(\sum_{y,z|_{\mathrm{ROI}}} 2\cos(\theta_{\mathrm{pa}}(y,z))\sin(\theta_{\mathrm{pa}}(y,z))\right)^2 + \left(\sum_{y,z|_{\mathrm{ROI}}} 2\left(\cos(\theta_{\mathrm{pa}}(y,z))\right)^2 - \sum_{y,z|_{\mathrm{ROI}}} 2\left(\sin(\theta_{\mathrm{pa}}(y,z))\right)^2\right)^2} \right) \\
&\overset{(2),(3)}{=} \frac{1}{2N_{\mathrm{ROI}}} \sqrt{4\left(\sum_{y,z|_{\mathrm{ROI}}} \sin(2\theta_{\mathrm{pa}}(y,z))\right)^2 + \left(\sum_{y,z|_{\mathrm{ROI}}} 4\cos(2\theta_{\mathrm{pa}}(y,z))\right)^2} \\
&= \frac{1}{N_{\mathrm{ROI}}} \sqrt{\left(\sum_{y,z|_{\mathrm{ROI}}} \sin(2\theta_{\mathrm{pa}}(y,z))\right)^2 + \left(\sum_{y,z|_{\mathrm{ROI}}} \cos(2\theta_{\mathrm{pa}}(y,z))\right)^2}
\end{aligned}
$$

□

The results presented in section 6 (main article) are based on the datasets presented in Fig. 11 showing the OAV-image in the 1st and the x-ray dark-field image in the 2nd column. PCA results of the entire scan are separated showing the results of the order parameter ω in the 3rd and the orientation axis in the 4th column. The STXM mask used for statistical evaluation is depicted in the 5th column. Special attention was drawn on masking single cells which is important for determination of the nematic order parameter s. Parameters of each scan are listed in Tab. 1.

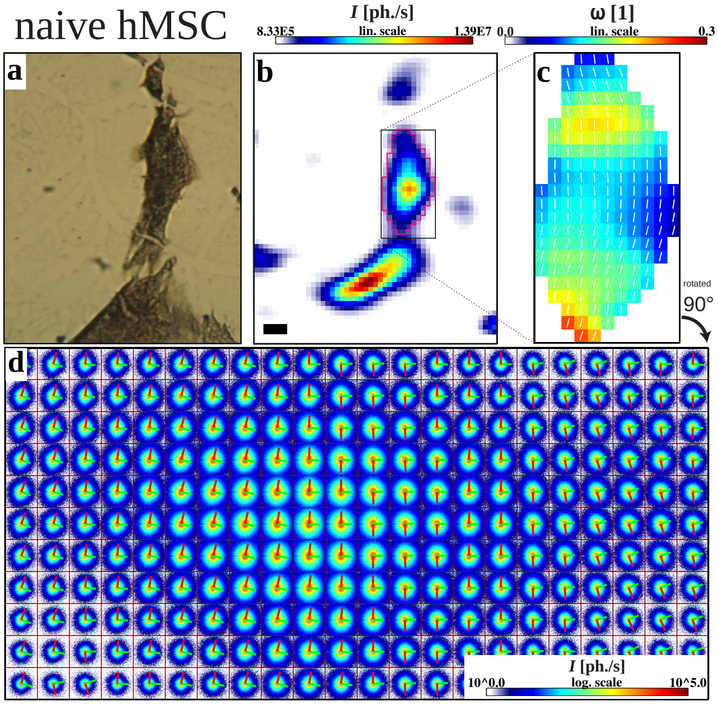

Figure 7: (a) OAV-image of freeze-dried naive hMSCs right before recording. (b) X-ray dark-field image using the micro-SAXS setup with a photon energy of $E_{\mathrm{ph.}}$=8.7 keV). Scalebar: 40 µm. (c) PCA results of the marked region after applying a STXM mask, indicated as a pink contour line in b. (d) Composite image of the scan area shown in c. By PCA, two basis vectors are computed for every diffraction image.

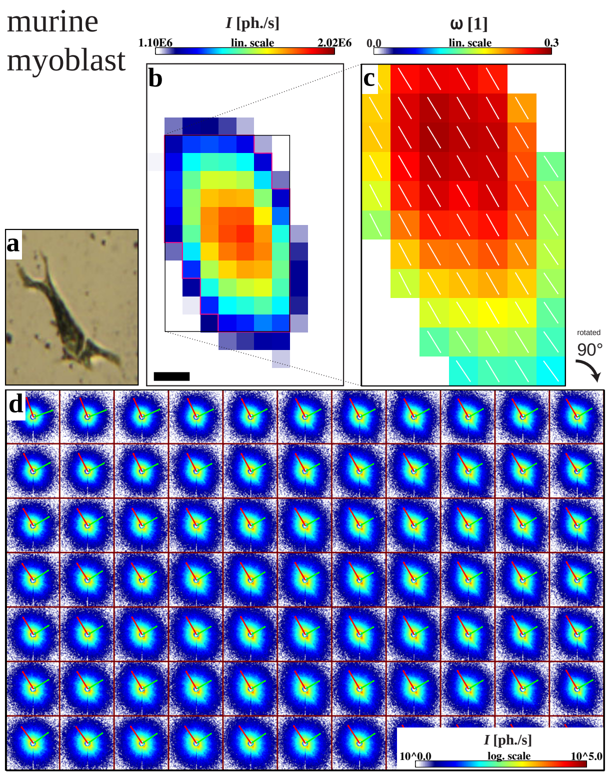

Figure 8: (*a*-*d*) equivalent arrangement as in Fig. 7, here on a murine myoblast. Scalebar: 10 µm.

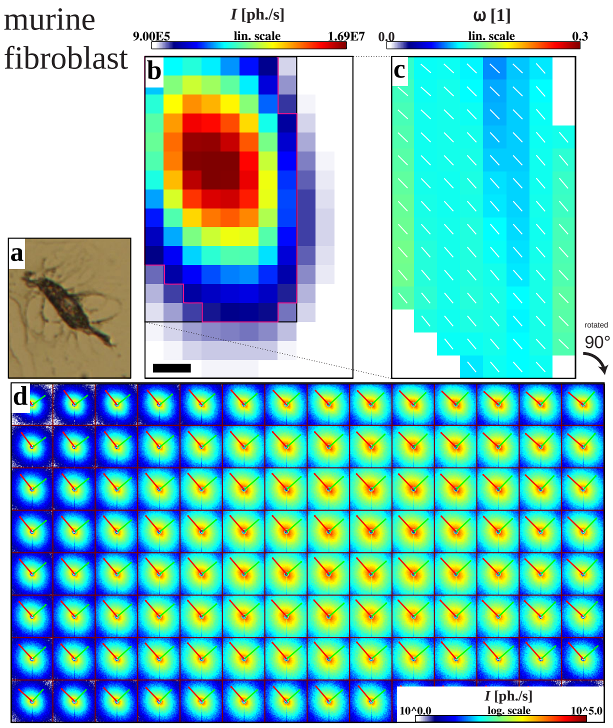

Figure 9: (*a*-*d*) equivalent arrangement as in Fig. 7, here on a murine fibroblast. Scalebar: 10 µm.

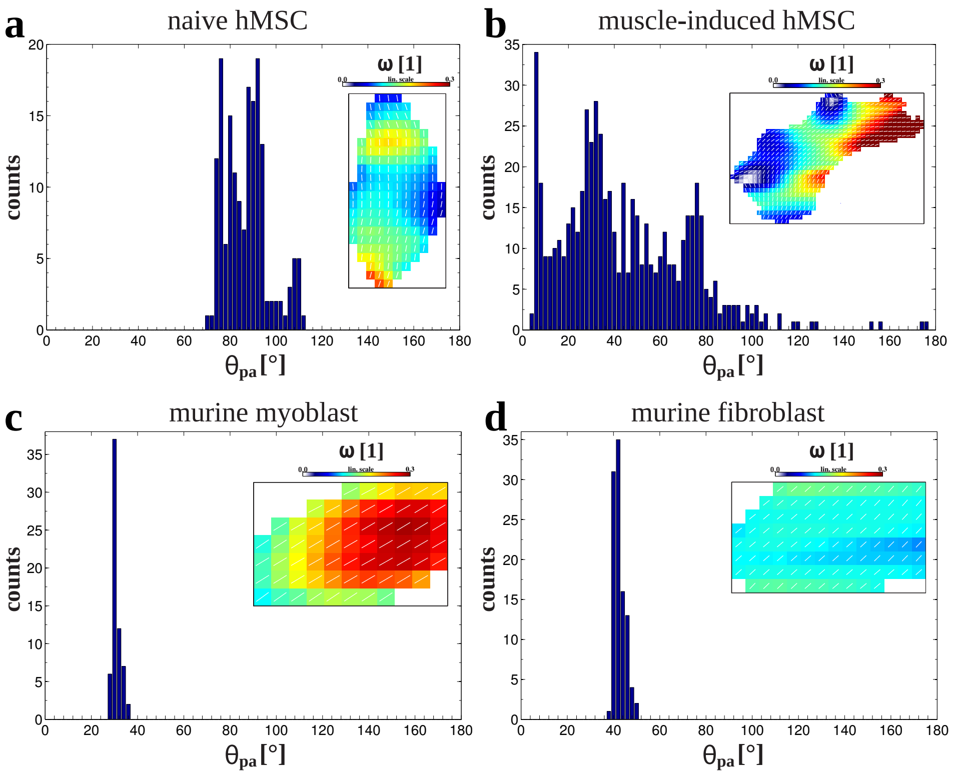

Figure 10: Histograms showing the distribution of structure orientation angles θ_{pa} in (*a*) a naive and (*b*) muscle-induced hMSC, (*c*) a murine myoblast and (*d*) a murine fibroblast.

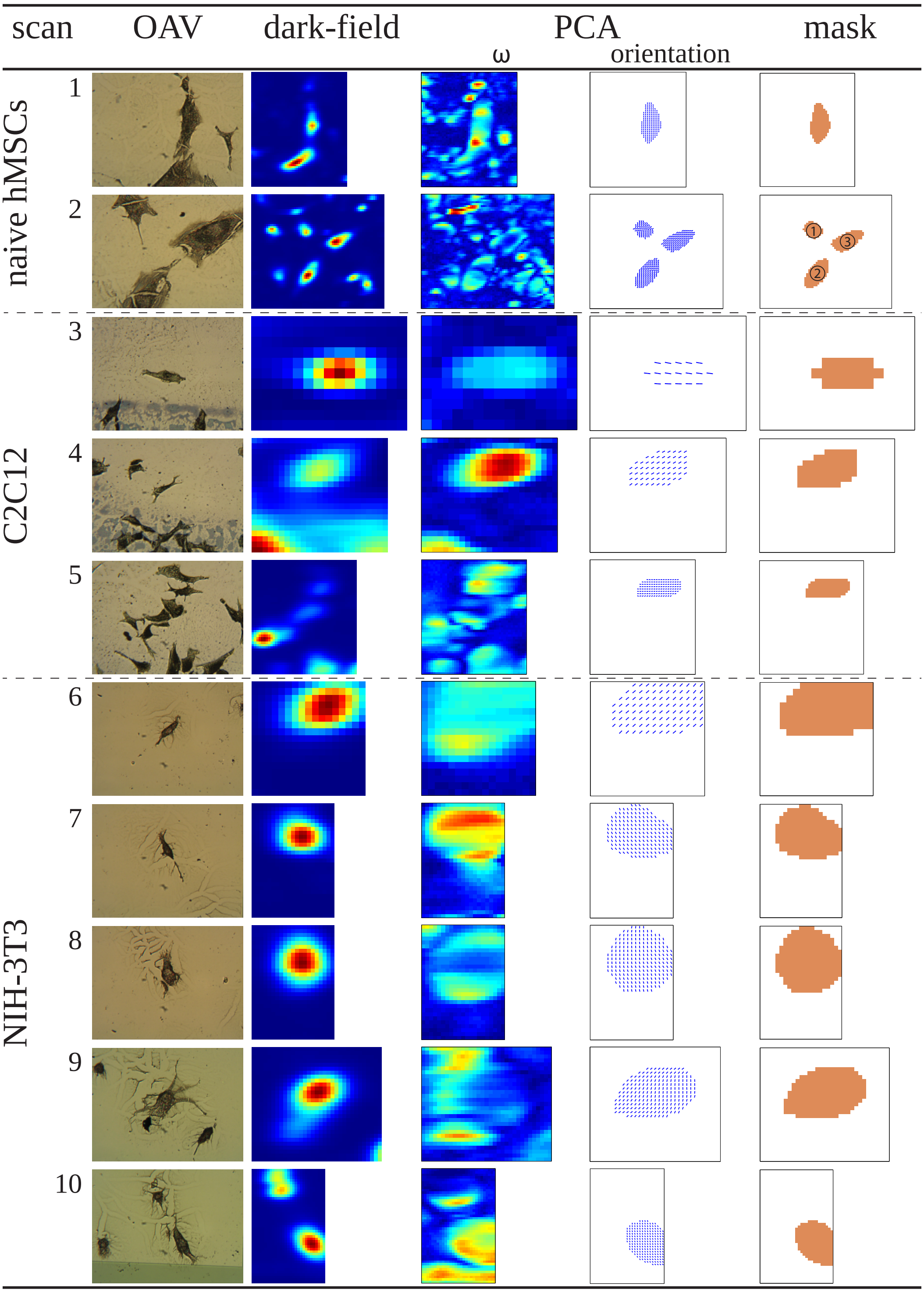
scan
OAV
dark-field
PCA
ω
orientation
mask
naive hMSCs
C2C12
NIH-3T3
1
2
3
4
5
6
7
8
9
10
①
②
③

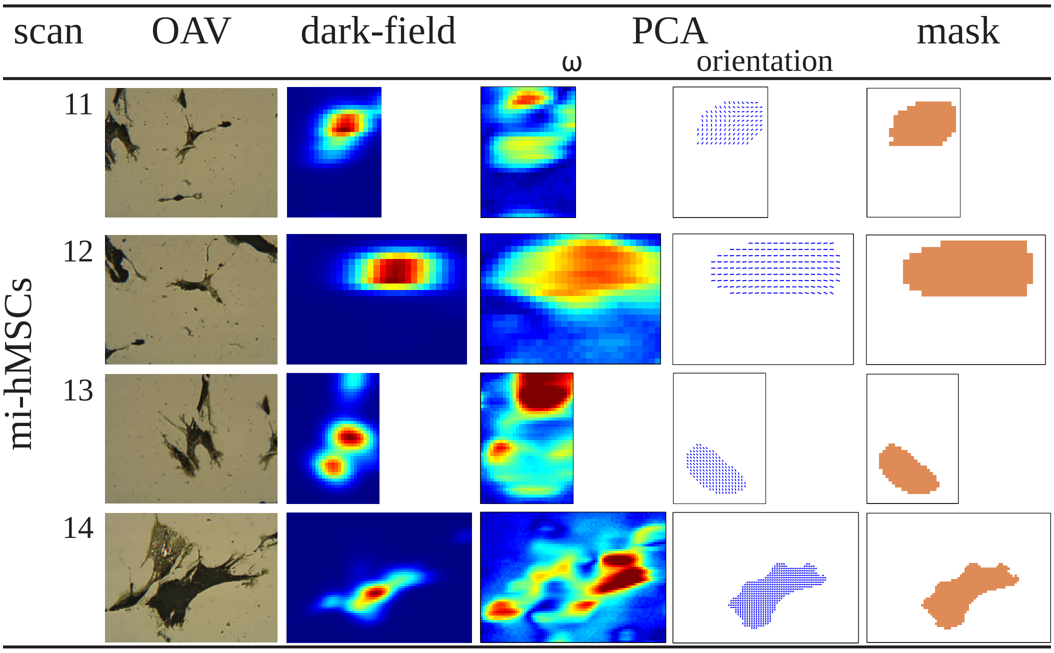

Figure 11: Overview of the datasets taken into account for statistical analysis showing the OAV-image in the 1st, the x-ray dark-field in the 2nd, the PCA order parameter and orientation in the 3rd and the 4th as well as the STXM masks for cells (red) in the 5th column. Note, that for presentation reasons individual limits were set for colorbars (not shown), so that a direct comparison between dark-fields is not applicable.

**cell type**	**naive hMSC**		**C2C12**			**NIH-3T3**					**mi-hMSC**			
**scan no**	**1**	**2**	**3**	**4**	**5**	**6**	**7**	**8**	**9**	**10**	**11**	**12**	**13**	**14**
Δ_y in µm	8	10	10	5	5	5	5	5	5	5	5	5	5	5
Δ_z in µm	8	10	10	5	5	5	5	5	5	5	5	5	5	5

Table 1: Scan step sizes for data shown in Fig. 7 (main article). All cells were grown on 1 µm-thick Si_3N_4-membranes, cryoprepared at alive state and then freeze-dried. All scans were performend at an energy of $E_{\text{ph.}} = 8.7\,\text{keV}$ with a dwell time of $t_{\text{dwell}} = 0.5\,\text{s}$ and a flux of $I_0 = 1.44 \cdot 10^{11}$ ph./s using the instrumentation of the cSAXS endstation.

11 Bibliography

References

1. O. Bunk, M. Bech, T. H. Jensen, R. Feidenhans'l, T. Binderup, A. Menzel, and F. Pfeiffer. Multimodal x-ray scatter imaging. *New Journal of Physics*, 11(12): 123016, 2009.

2. A. J. Engler, S. Sen, H. L. Sweeney, and D. E. Discher. Matrix elasticity directs stem cell lineage specification. *Cell*, 126(4):677–689, 2006.

3. B. Henrich, A. Bergamaschi, C. Broennimann, R. Dinapoli, E.F. Eikenberry, I. Johnson, M. Kobas, P. Kraft, A. Mozzanica, and B. Schmitt. Pilatus: A single

photon counting pixel detector for x-ray applications. *Nuclear Instruments and Methods in Physics Research Section A: Accelerators, Spectrometers, Detectors and Associated Equipment*, 607(1):247 – 249, 2009. Radiation Imaging Detectors 2008Proceedings of the 10th International Workshop on Radiation Imaging Detectors.

4. S. Kalbfleisch, H. Neubauer, S. P. Krüger, M. Bartels, M. Osterhoff, D. D. Mai, K. Giewekemeyer, B. Hartmann, M. Sprung, and T. Salditt. The Göttingen Holography Endstation of Beamline P10 at PETRA III/DESY. *AIPConf.Proc.*, 1365(1):96–99, 2011.

5. Lawrence Berkeley National Laboratory. The center for x-ray optics (cxro). last accessed on 04/01/15. URL `http://www.cxro.lbl.gov/`.

6. B. Liu, T. H. Besseling, M. Hermes, A. F. Demirors, A. Imhof, and A. van Blaaderen. Switching plastic crystals of colloidal rods with electric fields. *Nat Commun*, 5:3092, 2014.

7. T. Salditt, S. Kalbfleisch, M. Osterhoff, S. P. Krüger, M. Bartels, K. Giewekemeyer, H. Neubauer, and M. Sprung. Partially coherent nano-focused x-ray radiation characterized by Talbot interferometry. *Opt. Express*, 19(10):9656–9675, May 2011.

8. B. Weinhausen and S. Köster. Microfluidic devices for x-ray studies on hydrated cells. *Lab Chip*, 13:212–215, 2013.
